# Working from home and mental well-being at different stages of the COVID-19 pandemic

**DOI:** 10.1371/journal.pone.0312299

**Published:** 2024-11-13

**Authors:** Sandra M. Leitner

**Affiliations:** The Vienna Institute for International Economic Studies, Vienna, Austria; Asia University, TAIWAN

## Abstract

This paper analyses the relationship between working from home (WFH) and mental well-being at different stages during the first two critical years of the COVID-19 pandemic, when governments repeatedly imposed lockdowns and enacted WFH mandates to contain the spread of the virus. Using data from a representative survey conducted at four different time periods in 2020 (first lockdown, subsequent gradual reopening), 2021 (further lockdown) and 2022 (restrictions widely lifted) in the 27 EU member states, it examines the changing role of several mediators over time: work-family conflict, family-work conflict, stability, resilience, isolation, the importance of different support networks, workload, physical risk of contracting COVID-19 at work and housing conditions. For the first lockdown, it also differentiates by previous WFH experience, in terms of WFH novices and experienced WFH workers. It likewise differentiates by gender, in order to take the potential gendered nature of COVID-19 measures into account. The results point to several important mediators: for those who work from home, less family-work conflict and isolation, but greater stability, resilience, network support from family and friends, and superior housing conditions were associated with better mental well-being. The relevance of mediators was specific to certain stages of the pandemic. Stability was the most important mediator during the first lockdown. Work-family conflict and family-work conflict were only relevant during the first lockdown, while resilience and isolation mattered especially towards the end of the pandemic. Unlike established WFH workers, WFH novices had an advantage during the first lockdown, benefiting from lower family-work conflict and more helpful networks of family and friends. Our results differ by gender: for females who undertook WFH, important mediators were work-family conflict and family-work conflict. Both were related to adjustments they had to make in work and non-work hours in response to the enforced closure of schools and childcare facilities. For males who undertook WFH, support from networks of family and friends was an important mediator during the first lockdown.

## Introduction

During the COVID-19 pandemic, when many workplaces were obliged to close, working from home (WFH) became the norm for millions of workers. However, WFH can be seen as a double-edged sword. While it greatly enhances workers’ flexibility and work autonomy [1]–something that is recognised as improving both overall quality of life (including family life) [2] and productivity [3, 4]–it can also adversely impact workers’ mental health. Several empirical studies corroborate the health repercussions of WFH in terms of greater stress, depression and fatigue; but they also show that the intensity of WFH makes a difference [5–8]. The relationship between WFH and mental health is complex and is affected by many factors. The pre-pandemic literature shows that social isolation, extended working hours, unclear delineation and conflict between work and family, and lack of organisational support and interaction with colleagues are all important mediators associated with poorer mental health [9].

However, the COVID-19 pandemic created an unprecedented context not only for the expansion of WFH, but also for the psychological impact of WFH. First, social distancing measures and the repeated lockdowns put in place by governments to contain the spread of the virus forced many employees to work from home every day for an indefinite period, with little choice in the matter and often no time to prepare. Any form of mandatory WFH can lead to negative mental health outcomes. This was already indicated by the pre-pandemic literature [10], but has been further corroborated by the growing body of COVID-19-related literature, which largely finds that WFH had adverse effects on various mental health outcomes, such as well-being, stress, depression, fatigue and exhaustion [11, 12].

Second, the rapid spread and the severity of the COVID-19 pandemic increased fear of the disease. It has been shown that this led to adverse mental health outcomes, such as stress, anxiety and depression [13, 14]–mainly among essential and frontline workers who continued to go into work and interact face to face more frequently, rendering them more exposed to viral transmission [15–19]. In contrast, by providing a safe work option, WFH mitigated the fear of COVID-19 [20, 21] and its negative impact on mental health [22].

The new COVID-19 context affects not only the psychological impact of WFH, but also the role that different mediators play in the relationship between WFH and health-related outcomes. The pandemic literature highlights important new mediators specific to the COVID-19 situation, with repeated and lengthy WFH mandates. These came to be associated with worse mental health outcomes among those who worked from home either for longer or for a greater proportion of the week [23, 24]; who had to live and work in a crowded home or confined space [25]; who had an aversion to WFH [26]; or who felt more and more isolated and lonely due to long periods of WFH [27]. And just as before the pandemic, work-family/family-work conflict or a weakening of the social support networks with co-workers had an adverse impact on the mental well-being of those who worked from home [11].

However, longitudinal studies are rare in this strand of literature, and so little is known about the relationship between WFH and mental health or the potentially changing role of different mediators during the pandemic, both when countries underwent repeated lockdowns of varying degrees of severity in response to new waves of COVID-19, and when the context changed in 2021, as COVID-19 vaccines to protect against severe illness and death started to become available. Specifically, new waves of the virus prompted governments to repeatedly enact WFH mandates–often temporarily converted into WFH recommendations in the intervening periods–which forced workers to remain in, or return to, WFH for an extended and indefinite period. In this context, the scant longitudinal COVID-19 literature only focuses on short phases around specific pandemic events, such as the transition to WFH at the beginning of the pandemic or the effect of individual lockdowns (typically the first). For instance, it has been shown that people who switched to WFH during COVID-19 have worse mental health outcomes [28, 29] and a greater number of mental health issues than pre-WFH [30]. The home-office environment, the presence of children at home and reduced communication with co-workers have all been mooted as the key mediators leading to lower mental health levels [30]. For many, WFH was a new experience, often associated with worse mental health outcomes among WFH novices in terms of depression, work exhaustion or burnout [31]–at least initially [32], before they became accustomed to the new situation. However, in this literature, the role of potential mediators remains unexplored. Furthermore, the scant empirical evidence does suggest that the mental health situation of those people who worked from home also changed during the pandemic–but in different ways, depending on the specific lockdown and its subsequent lifting [33–35]. For instance, Somasundram and colleagues [33] show that between the second wave of COVID-19 in Canada and the end of the third, levels of stress and burnout among those working from home decreased significantly, and there is some evidence that this may be related to the greater support that they received from their colleagues. However, the mediating role of employee support has not been systematically analysed. Wels and colleagues [34] study three different stages of the pandemic in the UK (first lockdown in mid-2020; the easing of restrictions at the end of 2020; and the second lockdown at the end of 2021) and show that WFH was associated with greater psychological distress–but only during the second lockdown. However, there has been no analysis of the potential role of mediators. And the relevance of different mediators likewise changed [36]. Specifically, Wielgoszewska and colleagues [36] show–for three stages of the pandemic in the UK (first lockdown in mid-2020; the easing of restrictions immediately thereafter; and the third lockdown in early 2021) and on the basis of three different cohort studies–that loneliness was an important mediator that contributed to higher levels of anxiety and depression among those who worked from home, especially during the easing phase in mid-2020 and the third lockdown in 2021. In this context, the longitudinal study by Wood and colleagues [35] is particularly instructive and is closest to our study. It uses data from two four-week diary studies conducted in 2020 in the UK among homeworking university employees–in spring 2020 (when the UK was in lockdown) and in autumn 2020 (when the restrictions had been relaxed somewhat). It shows that greater work-family conflict, family-work conflict and divergence from normal work patterns were associated with worse mental health outcomes, but only during the first lockdown. By contrast, job insecurity was only relevant during the subsequent, more relaxed phase, and was also associated with worse mental health outcomes. Some mediators were of importance in both phases: loneliness–as an indicator of social isolation–was associated with poorer mental health outcomes (also in the study by Wielgoszewska and colleagues [36]), whereas greater job autonomy, social support and detachment from work were associated with better mental health outcomes. Other factors turn out to have been totally irrelevant, such as hours worked as a proxy for workload, technical/ICT constraints, or the fear of COVID-19.

Moreover, the context of the COVID-19 pandemic also changed from 2021, with the availability of COVID-19 vaccines that largely protected people from falling seriously ill, being hospitalised and dying. It has been shown that the vaccinations reduced fear of COVID-19 [37] and mental distress–particularly in vaccinated individuals, but also among those who had not been vaccinated [38, 39]. The importance of vaccinations in the WFH context is, however, limited [40], indicating that fear of COVID-19 infection was not a key source of psychological distress among WFH workers, but was more relevant in the case of essential and frontline workers.

Against such a backdrop, this paper contributes in three important ways to the growing literature on the effects of the COVID-19 pandemic on workers’ mental health. First, it sheds light on the potentially changing importance of various mediators across different stages of the COVID-19 pandemic, when countries underwent repeated lockdowns of varying degrees of severity. It tests various mediators, such as work-family conflict (WFC), family-work conflict (FWC), stability, resilience, isolation, the importance of different support networks (i.e. family and friends vs. institutional networks), workload, physical risk of contracting COVID-19 at work, and housing conditions. This is a longitudinal study–one of the very few. However, in contrast to the existing literature, which mainly looks at the first COVID-19 year [33–36], it takes a longer-term perspective and identifies the nexus between WFH and mental health–measured by the WHO-5 Well-Being Index–at different stages during the first two critical years of the pandemic: (i) April to June 2020, when most EU member states were in their first lockdown; (ii) June to July 2020, when economies and societies were gradually reopening; (iii) February to March 2021, when countries were again in various stages of lockdown; and (iv) March to May 2022, when many EU countries had already greatly reduced restrictions after the final lockdown in winter 2021/22. The study thus covers the second year of the COVID-19 pandemic, when vaccinations became widely available and many economies endured their very last lockdown.

Second, the paper highlights differences in the relevance of the various mediators, according to whether there was any previous experience of working from home–a consideration that has not previously received any attention in the literature. Specifically, in the context of the first lockdown, a distinction is drawn between (i) *WFH novices* with no prior experience; and (ii) *established WFH workers* with at least some previous experience of WFH. This distinction is important, since being forced to engage in WFH by the WFH mandate without being prepared for it caused additional stress and poorer mental health among workers [31, 32].

Third, the paper also differentiates by gender, in order to test whether the role of the mediators at different stages of the pandemic varies between men and women. This is something that until now has not been studied in a longitudinal setting. Gender is generally seen as an important factor contributing to stress, burnout and negative outcomes in WFH environments, because of the greater involvement of women in household and caregiving tasks. The pre-pandemic literature points to poorer mental health outcomes for women in WFH settings–mainly due to lower autonomy [41] and more work-family conflict [42–44]. The COVID-19 pandemic aggravated the situation of women who were either already working from home or had to transit to WFH when the closure of schools and childcare facilities forced them to assume additional childcare responsibilities. In fact, several studies show that most of the extra household and childcare work caused by COVID-19 devolved upon women [45–47]. During COVID-19, women were reported to have had poorer mental health outcomes than men [9, 48, 49]. Moreover, women who switched to WFH at the beginning of the pandemic had higher psychological distress levels [50] and a greater risk of developing new health problems [30]. The higher levels of psychological distress among women working from home were associated with the amount of time spent on housework and childcare [49], the greater number of hours worked and the lower degree of control they had over their working time [51].

Methodologically, the paper applies a structural equation modelling (SEM) approach–plus multigroup analysis across genders–to data from four rounds of Eurofound’s e-survey Living, Working and COVID-19, which captures the economic and social effects of the COVID-19 crisis across the EU at four different points in time during the pandemic. SEM provides a unified framework of analysis that allows us to identify the complex relationships between the different variables in our model; to measure the simultaneous influence of mediating variables on the relationship between WFH and mental well-being; to estimate our latent construct of well-being from multiple indicator variables; and to test the reliability and validity of the measures used. The e-survey is a representative dataset involving a large sample of workers in the 27 EU member states (as of 2020), rather than the small samples widely used in this strand of literature. The results are therefore representative of the average worker in the EU27. Our total sample consists of persons who were either employed or self-employed at the time of the survey, with sample sizes varying across the rounds.

Our findings suggest that–while there is no direct relationship between WFH and mental well-being–there are several important mediators whose importance changes over the two years under consideration. Stability is the only mediator that is relevant over the entire two-year pandemic period. WFC and FWC were only relevant during the first lockdown, while resilience and isolation only mattered in the second year of the pandemic, especially when most EU economies had lifted their restrictions. Some mediators were only available in certain survey rounds, but also turned out to be relevant (such as support from networks of family and friends, or housing conditions). Conversely, institutional networks, workload and the physical risk of contracting COVID-19 were not relevant mediators. We also find that WFH novices had an advantage over established WFH workers during the first lockdown, benefiting as they did from lower FWC and more helpful networks of family and friends. Moreover, our results differ by gender, highlighting the fact that males and females who worked from home were affected differently by the measures implemented at the various stages of the COVID-19 pandemic (such as lockdowns and reopening). For females who worked from home, important mediators were WFC and FWC, both of which were related to the adjustments they had to make in work and non-work hours in response to the enforced closure of schools and childcare facilities during the lockdowns (especially the first). For males who worked from home–and especially WFH novices–support from networks of family and friends was an important mediator.

The rest of the paper is structured thus: the following two sections discuss the data source and define the variables. The next two sections then lay out the theoretical framework and the methodological approach to determining the direct and indirect effects (through mediators) of WFH on workers’ mental well-being. The results are then presented and discussed in the subsequent section for both the total sample and separately by gender. The final section summarises our findings and sets out our conclusions.

## Data

The data for this study come from various rounds of Eurofound’s e-survey, Living, Working and COVID-19, which captures the economic and social effects of the COVID-19 crisis across the EU. The e-survey is particularly suited to this analysis, as it includes a set of questions that describe employees’ mental well-being; several questions on WFH before and during the pandemic; rich information on working conditions that can be used to construct different mediators; and worker characteristics (for more details, see the section on Measures).

To date, five rounds have been carried out: the first was conducted between 9 April and 11 June 2020, when most EU member states were in their first lockdown; the second was between 22 June and 27 July, when economies and societies were gradually reopening; the third was conducted one year into the pandemic, between 15 February and 30 March 2021, when countries were still at various stages of lockdown; the fourth round was a panel-only survey, where panel respondents were recontacted to track developments since the beginning of the pandemic; and the fifth round was conducted between 24 March and 2 May 2022 and looked at how life had changed over the previous two years. However, since the panel survey has not yet been completed, it is not available to researchers and therefore does not form part of the analysis, which uses rounds 1, 2, 3 and 5. The scope of the survey varies from round to round, and so the questionnaire (and consequently the set of mediators) also changes from round to round (see the section on Measures). This limits the comparability of the findings from the various rounds. The survey was conducted via the SoSciSurvey platform and was open to respondents from all countries aged 18 and over. In compliance with Regulation (EU) 2018/1725, a General Data Protection Regulation (GDPR) note was made available to the respondents. Among other things, this specified the procedure for data processing and detailed the protection of personal data and respondents’ rights to access, rectify, erase and/or port their personal data; to restrict or object to the processing of their personal data; and to withdraw their consent. However, the survey was mainly promoted in the EU, so the final dataset is only available for the 27 EU member states (as of 2020); this limits the scope for generalising our findings to other parts of the world. Respondents were mainly recruited using convenience sampling, specifically by online snowball sampling methods and social media advertisements; thus those without access to the internet and those who do not use any of the social media channels on which the e-survey was promoted were excluded from the sample. This non-probability method results in a non-representative sample; however, the composition of the sample was adjusted to be representative of the demographic profile of the EU27 as a whole and of each individual EU member state, by applying weights based on gender, age, education and self-defined urbanisation levels (and in the latest rounds also on employment status). This study performs secondary analysis of the fully and robustly anonymised data initially and lawfully collected by Eurofound, so that no ethics approval was necessary. The data were used in accordance with the purposes for which they were originally collected. The data are available from Eurofound on request. The questionnaire is mainly based on questions from Eurofound’s European Quality of Life Survey (EQLS) and the European Working Conditions Survey (EWCS), while other questions are new or have been adapted from other sources, such as the EU Statistics on Income and Living Conditions (EU-SILC). The questionnaire was developed in English, but the survey was made available and launched in 22 different languages.

The data were cleaned to remove partial interviews, interviews that were completed too quickly and those that contained contradictory answers; they were then weighted by age crossed with gender (in 12 age-gender combinations), urbanisation level (urban and rural, based on respondents’ own assessment) and education level (tertiary and non-tertiary). Probability weights are provided in the dataset and can be used for within- and cross-country analysis.

The sample size after cleaning varied from round to round: 67,392 in round 1; 23,702 in round 2; 45,269 in round 3; and 36,891 in round 5. The final sample of the present study includes all 27 EU member states covered in the first, second, third and fifth rounds of the e-survey, and includes those participants who were either employed or self-employed at the time of the survey (we excluded participants who were either unemployed or inactive at the time of the interview) and for whom complete information was available: 38,314 in round 1; 10,928 in round 2; 21,271 in round 3; and 17,031 in round 5. In each of the four rounds analysed, females represented between 55% and 70% of the total sample.

## Measures

*Working from home (WFH)* is captured by various indicators which, depending on the available questions and indicators, differ across the survey rounds. In all four rounds, WFH was measured by the location of work, using the following question: ‘*During the COVID-19 pandemic*, *where did you work*?’ A person was considered to be working from home if the answer was ‘Home’. In round 1, we could also identify those who had previous WFH experience: in response to the question ‘*Have you started to work from home as a result of the COVID-19 situation*?’, a *WFH novice* would answer ‘yes’ (with ‘no’ as the reference category). In response to the question ‘*How frequently did you work from home before the outbreak of COVID-19*?’, an established *WFH worker* would answer either (1) daily, (2) several times a week, (3) several times a month, or (4) less often, with (5) never as the reference category. Moreover, in rounds 2, 3 and 5 we can also observe the intensity of WFH, measured as the total number of hours worked from home and captured by the question(s): ‘*Last month*, *how many hours per week did you work on average*? *Out of these*, *how many hours did you work from home*?’ We calculate the ratio of the total number of hours of WFH to the total number of hours worked, and use that as a measure of intensity in the analysis.

*Mental well-being* is measured by the five items of the WHO-5 Well-Being Index (WHO-5), which is a short and generic global rating scale that gauges subjective mental well-being. Specifically, it measures how a respondent has been feeling over the previous two weeks, in terms of: ‘*I have felt cheerful and in good spirits*’, ‘*I have felt calm and relaxed*’, ‘*I have felt active and vigorous*’, ‘*I woke up feeling fresh and rested*’ and ‘*My daily life has been filled with things that interest me*’. All five measures use a seven-point scale, ranging from 0 (at no time) to 6 (all of the time). All five measures are available for all survey rounds and show high internal consistency, with Cronbach’s alpha above the minimum value standard of *α* = 0.70 [52], namely *α*_1_ = 0.881, *α*_2_ = 0.889, *α*_3_ = 0.900 and *α*_5_ = 0.899.

*Work-family conflict (WFC)* is defined as the extent to which the demands of work interfere with family life. It was measured by the questions ‘*How often in the last 2 weeks (last month) have you felt too tired after work to do some of the household jobs which needed to be done*?’ and ‘*How often in the last 2 weeks (last month) have you found that your job prevented you from giving the time you want to your family*?’ Both questions used a five-point scale, ranging from 1 (always) to 5 (never). We reversed the scales, so that a higher value indicated a higher level of conflict. The correlation between the two items was 0.826 for round 1, 0.861 for round 2, 0.857 for round 3 and 0.853 for round 5.

*Family-work conflict (FWC)* is defined as the extent to which the demands of time devoted to the family interfere with the performance of work-related responsibilities. It was measured using the following two questions: ‘*How often in the last 2 weeks (last month) have you found it difficult to concentrate on your job because of your family*?’ and ‘*How often in the last 2 weeks (last month) have you found that your family responsibilities prevented you from giving the time you should to your job*?’ Both questions used a five-point scale, ranging from 1 (always) to 5 (never), which we again reversed so that a higher value indicated a higher level of conflict. The correlation between the two items was 0.725 for round 1, 0.711 for round 2, 0.700 for round 3 and 0.716 for round 5.

*Stability* was measured using three questions that referred to job, accommodation and income stability, namely: ‘*How likely or unlikely do you think it is that you might lose your job in the next 3 months*?’; ‘*How likely or unlikely do you think it is that you will need to leave your accommodation within the next 6 months because you can no longer afford it*?*’*; and ‘*Thinking of your household’s total monthly income*: *is your household able to make ends meet*?’ The first two questions used a five-point scale, ranging from 1 (very likely) to 5 (very unlikely), while the last question used a six-point scale, ranging from 1 (with great difficulty) to 6 (very easily). For the four rounds analysed, Cronbach’s alpha was *α*_1_ = 0.650, *α*_2_ = 0.558, *α*_3_ = 0.615 and *α*_5_ = 0.601.

*Resilience* was measured as the extent to which respondents agreed or disagreed with the following two statements: ‘*I find it difficult to deal with important problems that come up in my life*’ and ‘*When things go wrong in my life*, *it generally takes me a long time to get back to normal*.’ Both questions used a five-point scale, ranging from 1 (strongly agree) to 5 (strongly disagree). For the four rounds analysed, the correlation between the two items was 0.727 for round 1, 0.740 for round 2, 0.756 for round 3 and 0.762 for round 5.

*Isolation* was measured as the extent to which respondents agreed or disagreed with the following two statements: ‘*I feel left out of society*’ and ‘*Over the last two weeks*, *I have felt lonely*.’ The first statement used a five-point scale, ranging from 1 (strongly agree) to 5 (strongly disagree), while the second employed a six-point scale, ranging from 1 (all of the time) to 6 (at no time). We reversed the scales so that a higher value would indicate a higher level of feeling isolated. Since the second item was not available in the first survey round, we used the construct only for rounds 2, 3 and 5. The corresponding correlation between the two items was 0.692 for round 2, 0.950 for round 3 and 0.706 for round 5.

*Networks* and the social capital embedded in different networks were measured as the support respondents would get in different situations. It was based on six different scenarios following the initial question: ‘*From whom would you get support in each of the following situations*? *For each situation*, *choose the most important source of support*.’ The six situations referred to (i) *‘If you needed help around the house when ill’*; (ii) *‘If you needed advice about a serious personal or family matter’*; (iii) *‘If you needed help when looking for a job’*; (iv) *‘If you were feeling a bit depressed and wanted someone to talk to’*; (v) *‘If you needed help in looking after your children’*; and (vi) *‘If you needed help with shopping*.*’* From the answer options, we constructed dummy variables, differentiating between two types of network: (1) *a network of friends and family*, where the most important source of support is either a member of the family/relative or a friend/neighbour or someone else who is not family or a relative; and (2) *an institutional network*, where the most important source of support is a service provider, institution or organisation (with ‘nobody’ as the reference category). Information on network support was only covered in the first round of the survey. The corresponding Cronbach’s alphas were *α*_1_ = 0.657 for networks of family/friends and *α*_1_ = 0.460 for institutional networks.

*Workload* was measured using the following three questions: ‘*Over the last 2 weeks*, *how often have you worked in your free time to meet work demands*?’; ‘*During the COVID-19 pandemic have your working hours*: *(1) decreased a lot*, *(2) decreased a little*, *(3) stayed the same*, *(4) increased a little*, *(5) increased a lot*?’; and ‘*[Do] you [currently] have enough time to get the job done*?’ The first and the last question used a five-point scale, ranging from 1 (always) to 5 (never). We reversed the scale for the first question, so that a higher value indicated a more frequent need to work in one’s free time in order to meet work demands. All three questions were only asked in round 3, with a corresponding Cronbach’s alpha of *α*_3_ = 0.584.

*Physical risk* was measured by the three questions: ‘*In your work*, *are you currently in direct physical contact with people (colleagues*, *customers*, *passengers*, *pupils*, *patients*, *etc*.*)*?’; ‘*Do you think you are currently at risk of contracting the COVID-19 virus because of your job*?’; and ‘*For your job*, *are you required to wear personal protective equipment to prevent the spread of COVID-19*?’ While the first question used a five-point scale, ranging from 1 (always) to 5 (never), the latter two questions used two value labels, 0 (no) and 1 (yes). We reversed the scale of the first question, so that a higher value indicated a higher level of physical contact with people. All three questions were only asked in round 2. Cronbach’s alpha was *α*_2_ = 0.649.

*Accommodation* refers to the condition of the respondent’s accommodation and was measured by how problematic respondents considered six issues to be regarding their accommodation: (i) ‘*Lack of space in the home*’; (ii) ‘*Poor insulation/energy efficiency*’; (iii) ‘*Poor internet connection*’; (iv) ‘*No access to balcony/terrace/garden*’; (v) ‘*Noise from neighbours*’; and (vi) ‘*Noise from traffic*’. All six measures used a five-point scale, ranging from 1 (not at all problematic) to 5 (extremely problematic). This issue was only addressed in round 5. Cronbach’s alpha was *α*_5_ = 0.669.

Furthermore, in the analysis we included an additional set of controls: sex (in terms of female and ‘other’, with male as the reference category); the log of age and its square; the highest level of education (ISCED-11 based) classified into low (ISCED-0 to ISCED-02, as the reference), medium (ISCED-03 and ISCED-04) and high (ISCED-05 and above); the number of dependent children in the household; whether or not the partner lives in the same household (with single as the reference category); whether a person is self-employed or works part time (with full-time work as the reference category); whether a person lives in an urban region (defined as a medium to large town or city or city suburb, as against the open countryside or a small village/town); and sector of economic activity (NACE rev. 2, 1-digit). The sector of economic activity was not covered in round 1 of the e-survey, while occupational information was not captured in any of the e-survey rounds. From the Oxford Coronavirus Government Response Tracker (OxCGRT) project, which collected information on policy measures to tackle COVID-19 over the years 2020 to 2022, we also included three measures that were available for all countries in our sample (see https://www.bsg.ox.ac.uk/research/covid-19-government-response-tracker). First, the Stringency Index, which captures the stringency of lockdown policies: this is a composite measure of various closure and containment measures (i.e. school closures, workplace closures, cancellation of public events, restrictions on public gatherings, suspension of public transport, stay-at-home requirements, public information campaigns, restrictions on internal movement and international travel controls) and is available on a daily basis, with values ranging from 0 (no response) to 100 (most stringent response). Second, the Economic Support Index, which is an overall measure of financial assistance to households in terms of income support or debt/contract relief for households: it is also available on a daily basis, with values ranging from 0 (no support) to 100 (maximum support). And third, the number of total COVID-19 cases per million inhabitants, taken from the ‘Our World in Data’ site (downloadable at https://github.com/owid/covid-19-data/tree/master/public/data); this information is collected from the WHO Coronavirus Dashboard and is available on a daily basis (see https://covid19.who.int/data). All three measures were merged with the e-survey data using the exact date on which the interview took place, and are therefore reflective of the severity of the COVID-19 crisis and policy measures in place at the time of the interview.

In the analysis, we use probability weights, as provided in the dataset.

## Theoretical framework

We rely on the Job Demands-Resources (JD-R) model [53–55] for our general framework. It assumes that job characteristics can be classified as either ‘job demands’ or ‘job resources’, taking into account a large range of job characteristics, as well as conflict between work and family. Job demands refer to the physical, psychological, social and organisational characteristics of the job that require continued physical and/or mental effort or skills on the part of workers, and are therefore associated with physiological and psychological costs. Conversely, job resources refer to physical, psychological, social and organisational characteristics of the job that contribute to the achievement of work goals, help to reduce the physiological and psychological costs of job demands, and stimulate employees’ personal growth and development [56]. Originally focused on predicting outcomes of burnout and work engagement, the JD-R model has also been shown to be related to several dimensions of well-being [57]. We identify demands and resources by how they are rated by individuals–demands are rated negatively, while resources are rated positively [58]. We also assume that factors with a negative value have a negative impact on well-being, while factors with a positive value have a positive effect on well-being.

In this context, we postulate that WFH affects mental well-being both directly and indirectly, via working conditions: it affects the nature of working conditions by intensifying some of the demands placed on people and resources, while making other things easier. Furthermore, we assume that these demands and resources in turn have an impact on mental well-being.

In the choice of mediators, we are guided by the pre-pandemic and pandemic literature. This shows that (among other factors) extended working hours, social isolation, conflict between work and family, lack of organisational support, having to live in a crowded home or a confined space, lack of resilience and job insecurity are all associated with poorer mental health among WFH workers [9, 11, 25, 35, 36]. We also test mediators that have so far not received any attention in the literature. These include the physical risk of contracting COVID-19 (thought to cause stress and anxiety, and therefore to be negatively associated with mental well-being) and the role of different social network supports outside work (thought to be positively associated with mental well-being). We use all mediators available in each of the e-survey rounds to test the most complete model possible for each round.

## Methodological approach

In this analysis, we proceed in two steps. First, we conduct exploratory and confirmatory factor analysis (CFA) to assess the properties of the different latent constructs. We use factor loadings to shed light on the dimensionality of each construct and to identify the most relevant items of each construct; and we calculate Cronbach’s alpha (a measure of internal consistency or reliability), in order to determine how closely related the underlying items are that comprise each construct. Items with low factor loading and/or which show little internal consistency with the other items in the group are removed from each construct.

**Fig 1:**
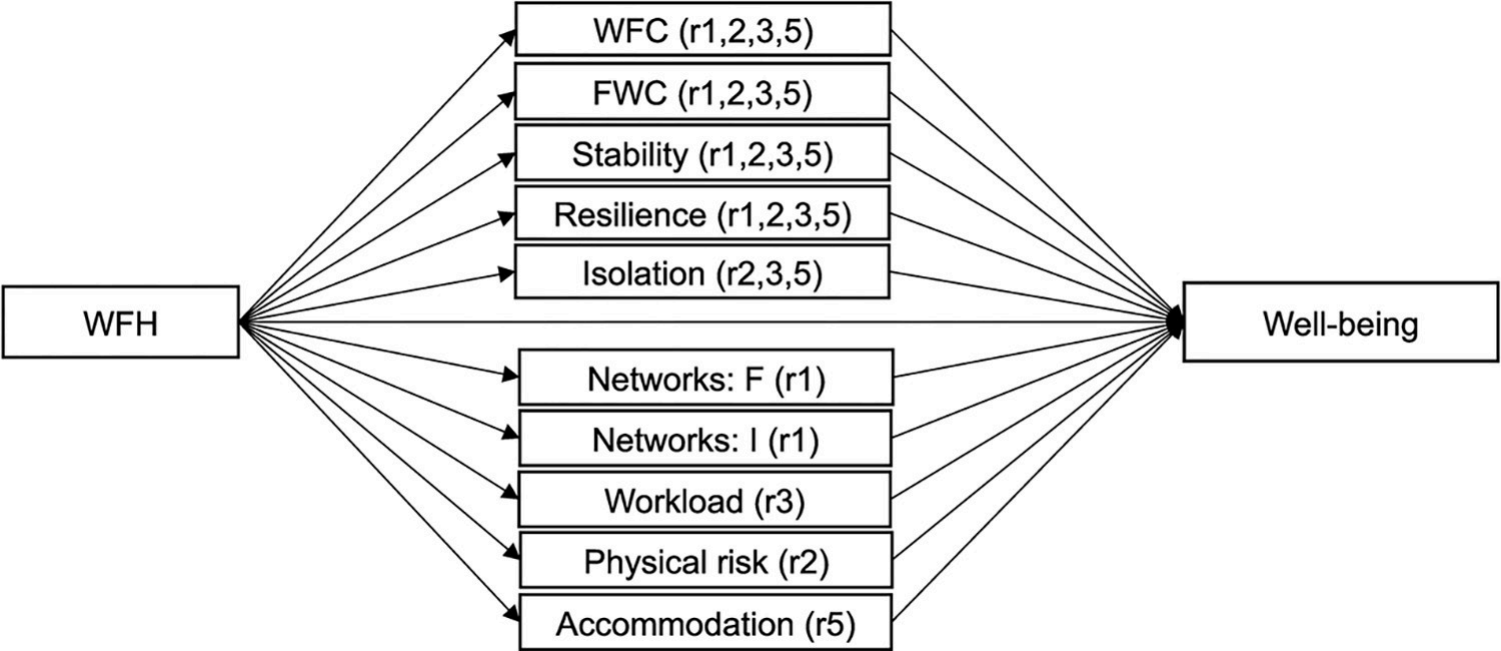
Conceptual model. Note: F = family and friends, I = institutions.

Second, we then use ‘purified’ scales in our SEM approach. Generally, SEM is used to identify complex relationships that involve multiple dependent and independent variables (mediators and outcomes) within a unified framework of analysis. Hence, it allows us to test the complex relationship between the different variables in our model; to measure the simultaneous influence of mediating variables on the relationship between WFH and mental well-being; to estimate latent constructs from multiple indicator variables; and to test the reliability and validity of the measures used. As is shown in Fig 1 below, in the model WFH (and different variants thereof) was included as an exogenous variable. WFC, FWC, stability, resilience, isolation, networks of friends and family, institutional networks, workload, physical risk and housing conditions were treated as mediators between WFH and mental well-being. We consider and test both the direct and the indirect relationships–through the various mediators–between WFH and mental well-being. This shows not only the importance of the different mediating channels by which WFH affects well-being, but also whether there is a separate direct effect between WFH and well-being, even after controlling for the different mediators. Mental well-being was treated as an endogenous outcome variable. All the mediators were used as composite measures, calculated from the underlying items of each construct and normalised to have a mean of zero and standard deviation of one to ease interpretation. Only the endogenous outcome variable was treated as a latent construct in the SEM estimations, with its own unique variances and error terms (*ε*_1_−*ε*_5_) (assumed to be normally distributed) for each of the WHO-5 items (*x*_1_−*x*_5_). We used a parallel multiple mediation model, where indirect effects are included simultaneously. The simultaneous inclusion of indirect effects allows us to compare the relative size of the effects, while eliminating the problem of estimation bias that occurs when multiple mediators that are intercorrelated are tested individually in simple mediation models [59]. For statistical inference, we use bootstrapping–with 1,000 replacements–to generate standard errors (and confidence intervals) of the products of model parameters [60]. Bootstrapping takes better account of the irregularity of the sampling distribution of the indirect (product) term, and therefore provides more accurate inferences about the indirect effects and the presence of mediation. Our conceptual model (as shown in Fig 1) can be translated into the following structural model:

$$M_{kijc}=\alpha_{k}+\beta_{k}{WFH}_{ijc}+\beta_{kz}X_{zijc}+\theta_{kj}+\omega_{kc}+\varepsilon_{kijc}\;for\;all\;k=1\;to\;K$$ (1)

$${WB}_{ijc}=\alpha_{0}+\gamma_{1}{WFH}_{ijc}+\beta_{z}X_{zijc}+\sum_{k=1}^{K}\pi_{k}M_{kijc}+\theta_{j}+\omega_{c}+\epsilon_{ijt}$$ (2)

where *M*_*kijc*_ in Eq (1) represents the *k* different mediators (i.e. WFC, FWC, stability, resilience, isolation, networks of friends and family, institutional networks, workload, physical risk and housing conditions) of individual *i* in industry *j* and country *c*, which are determined by, among other things, *WFH*_*ijc*_. As outlined above, the mediators differ across survey rounds, due to changes in the questionnaire from round to round of the survey. *WFH*_*ijc*_ refers to different WFH indicators, such as: (i) *WFH* (based on the location of work: home); (ii) WFH *novice* versus *established* WFH worker (differentiated by previous WFH experience); and (iii) *WFH intensity* (measured as the ratio of the total number of hours spent working from home to the total number of hours worked in a given time period). *WB*_*ijc*_ in Eq (2) refers to the WHO-5 Well-Being Index, which is determined by, among other things, the various mediators (*M*_*kijc*_). Hence, *WFH*_*ijc*_ is modelled both directly (Eq (2)) and indirectly, through the various mediators *M*_*kijc*_ (Eq (1)). In both equations, *X*_*zijc*_ is a matrix of *z* additional individual characteristics (such as sex, the log of age and its square, the highest level of education, marital status, the number of dependent children in the household, whether or not the partner lives in the same household, whether the respondent is self-employed or works part time, and level of urbanisation), while *μ*_*o*_ refers to industry fixed effects and *ω*_*c*_ to COVID-19-related policy measures and measures to deal with the severity of the health crisis (Stringency Index, Economic Support Index, total number of COVID-19 cases per million inhabitants). *ε*_*kijc*_ and *ϵ*_*ijt*_ are the error terms, assumed to be uncorrelated and to follow a multivariate normal distribution. We use the same specification and structure for each group in our joint multigroup analysis, but always exclude the relevant group variable (i.e. gender) from the list of additional control variables.

Owing to the linear nature of the constructs, we use the SEM approach, as implemented in Stata (version 15.1), which employs standard ordinary least squares (OLS) regressions for all dependent variables and fits SEMs via maximum likelihood.

As highlighted above, we apply probability weights (as provided in the dataset) to account for the sampling design and to make the results more representative of the population. As a consequence, however, it is no longer feasible to employ the standard absolute and relative fit indices that are typically used to assess model fit–such as the *χ*^2^ Goodness-of-Fit Statistic, the Root Mean Square Error Approximation (RMSEA), the Comparative Fit Index (CFI) or the Tucker-Lewis Index (TLI)–since the maximum likelihood assumption of independent observations is violated. Instead we use the Standardised Root Mean Square Residual (SRMR) and the Coefficient of Determination (CD) to establish the model fit. Cutoff values for the various measures are discussed in Hu and Bentler [61]. The former measures how closely the model comes to reproducing each correlation, on average, with a recommended value of less than 0.08. The latter is an overall summary of how well the model fits, and has a maximum value of 1.0. It should, however, be noted that the CD is sensitive to the inclusion of additional variables [62].

We should point out here that our analysis is subject to certain limitations. First, it is correlational in nature. While the SEM approach indicates the possible direction of effects, the cross-sectional nature of our study does not allow us to draw conclusions about causal relationships between variables. Second, the cross-sectional nature of our study also means that we do not account for reverse causality (endogeneity) between the different variables: that is best handled using time series data. Since the panel-only survey was not yet available to researchers at the time of writing, analysis of it is left for future research. We do recognise that reverse-causal relationships could exist between our study variables, and that would potentially introduce bias into our estimates. Third, due to changing questionnaires, not all mediators are available in all survey rounds–something that makes comparison across time difficult. Fourth, given the 1–4 week recall periods, there may be recall bias in our self-reported measures, which could affect the accuracy of our findings. Fifth, differentiating between WFH novices and experienced WFH workers may introduce bias into our estimates, as the experience of WFH may influence the perceived impact on mental well-being.

## Results

**Fig 2:**
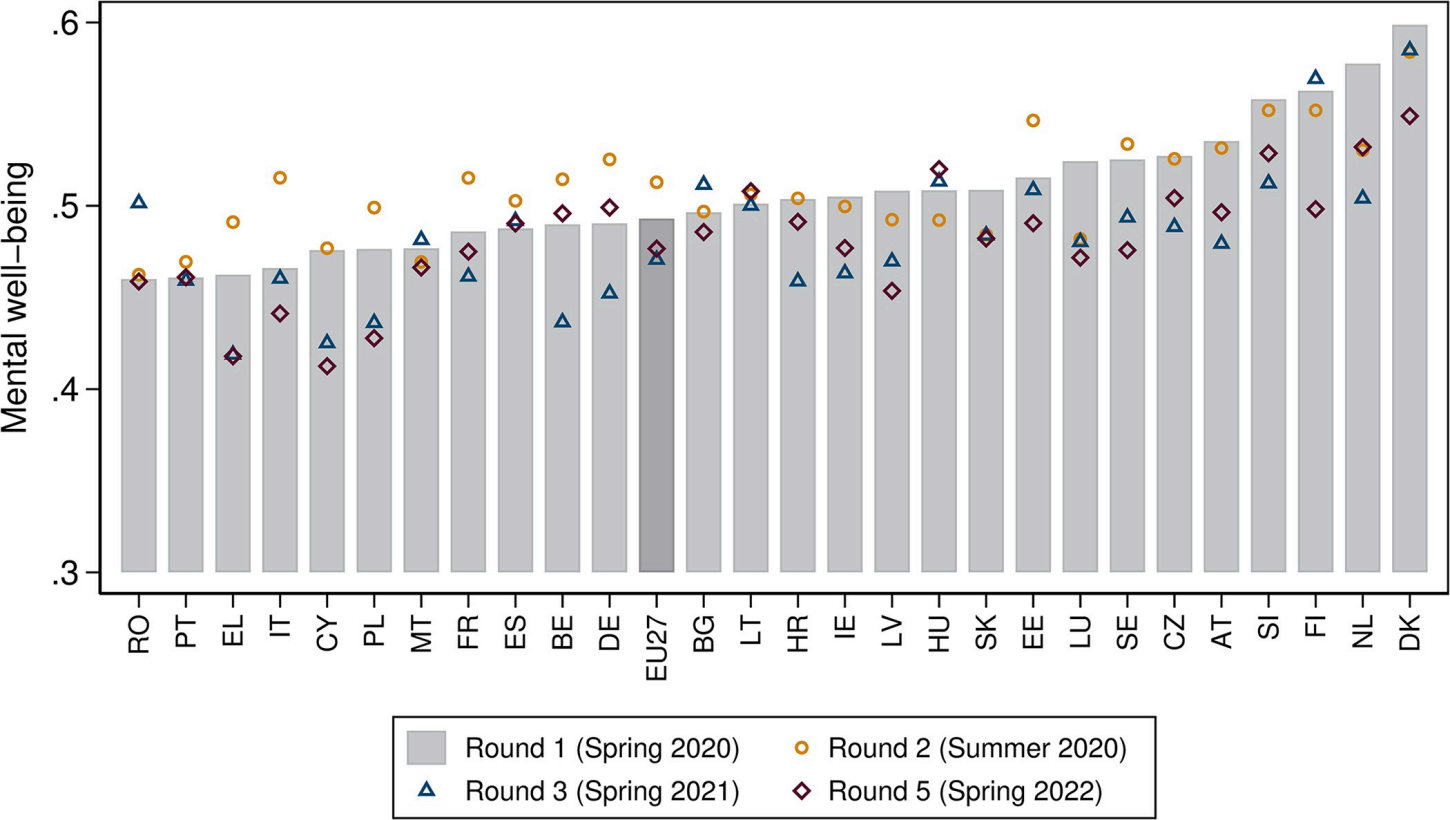
Mental well-being across EU member states and survey rounds. Note: The weighted means of the mental well-being index–standardised to lie between 0 and 1 –are shown for the final samples used in the analysis. Round 1 was conducted in spring 2020 (between 9 April and 11 June 2020), round 2 in summer 2020 (between 22 June and 27 July), round 3 in spring 2021 (between 15 February and 30 March 2021) and round 5 in spring 2022 (between 24 March and 2 May 2022). Round 1 indices–represented by the grey bar–are in ascending order; the darker grey bar refers to the EU27 aggregate. Source: Living, Working and COVID-19 (Eurofound), own calculations.

Fig 2 below shows the weighted average mental well-being index of the final survey samples used in the analysis for each survey round and for each EU member state–plus an EU27 aggregate. To ease interpretation, the index has been standardised to lie between 0 and 1.

Fig 2 points to a non-negligible heterogeneity in mental well-being across the EU member states during the first lockdown (round 1), which is also reflective of the country-specific context and the policy decisions taken. Mental well-being was lowest in Romania and the three Southern EU member states of Portugal, Greece and Italy. By contrast, mental well-being was highest in Denmark, followed by the Netherlands, Finland and Slovenia.

During the subsequent easing phase (round 2), things improved mainly in those member states that initially had lower mental well-being levels–most notably in Italy, which had been hard hit by the first COVID-19 wave and was the first EU member state to impose restrictions and enter strict lockdown.

With very few exceptions, mental well-being deteriorated thereafter, as captured by rounds 3 and 5: at the time of round 3, many member states were in another lockdown; and by the time of round 5, many of them had already greatly moderated the restrictions following the final lockdown in winter 2021/22. However, there is no clear pattern concerning the change between these two rounds. In some member states, mental well-being remained unchanged between rounds 3 and 5 (i.e. Portugal, Greece, Spain and Slovakia). Mental well-being deteriorated further in about half of the remaining member states, while it improved in the other half. However, in only a few EU member states (i.e. Belgium, Germany, Hungary and Lithuania) had mental well-being returned to, or even surpassed, the original level recorded at the time of the first lockdown (round 1).

In the EU27, mental well-being improved slightly between rounds 3 and 5, but remained below the initial mental well-being level observed during round 1.

**Fig 3:**
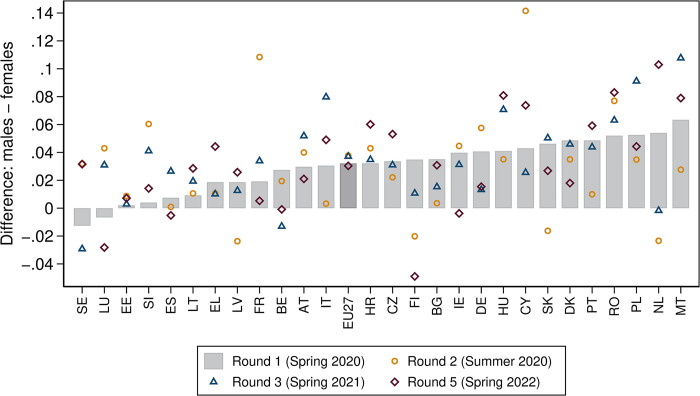
Gender mental well-being differential across EU member states and survey rounds. Note: The difference in the weighted means of the mental well-being index–standardised to lie between 0 and 1 –between males and females is shown for the final samples used in the analysis. Positive values indicate a mental well-being advantage among males over females. Round 1 was conducted in spring 2020 (between 9 April and 11 June 2020), round 2 in summer 2020 (between 22 June and 27 July), round 3 in spring 2021 (between 15 February and 30 March 2021) and round 5 in spring 2022 (between 24 March and 2 May 2022). Round 1 indices–represented by the grey bar–are in ascending order; the darker grey bar refers to the EU27 aggregate. Source: Living, Working and COVID-19 (Eurofound), own calculations.

Fig 3 shows the difference between males and females in the weighted (and standardised) average mental well-being index across EU member states–plus the EU27 aggregate–for each survey round. Positive differentials indicate better mental well-being among males than among females.

For the first lockdown phase (round 1), Fig 3 shows that–except for in Sweden and Luxembourg–mental well-being was always higher among males than among females. The discrepancy was particularly pronounced in Malta, the Netherlands, Poland and Romania.

During the subsequent COVID-19 phases (rounds 2 to 5), the mental well-being differential shifted quite a bit in all member states. One notable exception was Estonia, where very little change was observable. However, by and large, it remained positive. There are a few exceptions, though. For instance, during the easing phase after the first lockdown (round 2), the mental well-being differential reversed in some member states (Latvia, Finland, Slovakia and the Netherlands), indicating that females had better mental well-being than males. But this was only temporary, and a return to a positive mental well-being differential was observable when many member states were in another lockdown (round 3).

The mental well-being differential was also positive in most member states once restrictions had largely been lifted, following the final lockdown (round 5). In many countries, the differential exceeded the original level of the first lockdown phase (round 1); this was especially true of Greece, Hungary and the Netherlands. By contrast, in a few member states, the differential turned negative–e.g. Finland, Spain, Ireland and Belgium.

The broad variation in the mental well-being differential between the genders across member states and COVID-19 phases is barely reflected in the fairly stable EU27 differential, which increased slightly during the easing phase after the first lockdown (round 2) and in the subsequent lockdown phase (round 3), but then fell slightly below the initial level once restrictions were largely lifted following the final lockdown (round 5).

### Measurement model

The constructs in our model were measured using between two and five indicators, depending on the availability of data across the rounds. To ensure comparability, the factor structure of all the constructs used in the study is the same across all survey rounds.

S1 Table presents the means, standard deviations, Cronbach’s alpha coefficients (or correlation coefficients in the case of two-item constructs) and intercorrelations of the key variables used in the analysis, separately for each survey round. For the sake of brevity, information is only reported for the key variables used in the model (the full tables, including all individual control variables, are available from the author on request). It shows that for mental well-being, all alpha values are around 0.9. For the remaining variables, the alpha values are close to the minimum value standard of *α* = 0.70 [52], except for institutional networks (*α* = 0.46); this suggests that the construct has relatively little internal consistency and that results should therefore be interpreted with caution. We made several attempts to improve this construct’s internal consistency, but neither the exclusion of items currently considered nor the inclusion of alternative items proved successful. The correlation coefficients show that WFH is positively and significantly related to WFC, stability and resilience across all rounds; also to networks of friends and family and institutional networks for round 1; and to workload for round 2. By contrast, it is negatively and significantly related to FWC and isolation across all rounds; also to physical risk for round 2; and to accommodation for round 5. Moreover, the correlation between the latent construct of mental well-being and all mediators used in the analysis–transformed into composite measures–is highest for resilience (ranging between 0.41 and 0.55) and isolation (ranging between -0.56 and -0.52) in all rounds. The remaining correlations range between -0.08 and 0.37, demonstrating adequate discriminant validity.

S2 Table reports all the construct items with the corresponding standardised and unstandardised factor loadings. For constructs with only two variables, there is an empirical under-identification issue, since at least three variables are needed per latent construct to calculate all parameters (loadings and error variances) and standard errors. Hence, in this case, only the standardised factor loadings are reported: these are taken from a measurement model that includes all constructs at once, in order to make use of the degrees of freedom from other constructs. It shows that loadings for all items are statistically highly significant at the 1% level and are sizeable, predominantly lying above 0.5.

A multifactor confirmatory factor analysis model of the measurement model, with health as the only latent construct, showed good model fit across the survey rounds: SRMR = 0.032 and CD = 0.889 for round 1; SRMR = 0.032 and CD = 0.889 for round 2; SRMR = 0.026 and CD = 0.902 for round 3; and SRMR = 0.027 and CD = 0.902 for round 5.

However, before undertaking SEM estimations, the issue of invariance–specifically, *time-invariance*–needs to be addressed. Since some of the same constructs (i.e. mediators and latent constructs) appear repeatedly in our analysis, we need to make sure that they are equivalent across the survey rounds, and therefore that they measure the same concepts and are equally interpretable. For this purpose, we use a covariance structure analysis method to examine time-invariance in reliability in multi-wave, multi-indicator models [63]. It proceeds stepwise and first specifies a reference CFA model with time-invariant factor loadings across rounds (and zero means); this allows for free error-covariances across rounds. As a second step, equality of the covariances of error terms across rounds is introduced.

The restrictions could be evaluated using the *χ*^2^ difference test, with a statistically significant decrease in *χ*^2^ for the more constrained model, indicating non-invariance. However, since we use weights, *χ*^2^ statistics are not available. Hence, in addition to the SRMR and the CD, we also perform adjusted Wald tests and report p-values from the null hypothesis of equality of the covariance of error terms across time.

The invariance of the measures across the rounds was analysed as far as possible. Specifically, factorial invariance was tested across all four waves for mental well-being and stability. The other constructs are either composed of only two items (and are therefore subject to under-identification–e.g. WFC, FWC, resilience and isolation), or else were used only once and were therefore not tested for time-invariance (such as networks of friends and family, institutional networks, workload, physical risk and the housing conditions).

For the latent construct of mental well-being, the first step yields the following statistics: SRMR = 0.031 and CD = 0.893; the second step yields the following statistics: SRMR = 0.033 and CD = 0.894, indicating a slight increase in the SRMR. The respective p-value from the Wald test is equal to 0.0968, indicating that for health, the null hypothesis of equality of the covariance of error terms across time is rejected, but only at the 10% level of statistical significance.

As concerns stability, the first step yields the following statistics: SRMR = 0.016 and CD = 0.648 and the second step the following statistics: SRMR = 0.021 and CD = 0.646, also indicating a slight increase in the SRMR. The respective p-value from the Wald test is equal to 0.1174, indicating that for stability the null hypothesis of equality of the covariance of error terms across time cannot be rejected at conventional levels of statistical significance.

### Structural equation modelling

The results of the empirical analysis are discussed in the next two sub-sections. The first reports the direct and indirect (mediated) effects of WFH on mental well-being for each survey round, while the second reports the direct and indirect (mediated) effects of WFH on mental well-being by gender, in the context of a multigroup analysis.

#### Direct and indirect effects of WFH on mental health

The direct and indirect effects of WFH on mental well-being using a bootstrap bias-corrected method with a 95% confidence interval are reported in Table 1 below. The results are presented for two different concepts of WFH. Columns (1)-(6) refer to results when WFH is defined as a binary variable in terms of whether or not a person worked from home during the pandemic; columns (1), (4), (5) and (6) refer to the findings for rounds 1, 2, 3 and 5, respectively, while columns (2) and (3) refer to round 1, when WFH is further distinguished by workers’ previous experience of WFH, in terms of WFH novices (i.e. those without any previous experience of WFH) and established WFH workers (i.e. those with prior WFH experience). Columns (7)-(9) refer to the results for rounds 2, 3 and 5, respectively, when the frequency of WFH is used instead (defined as the ratio of the total number of hours working from home to the total number of hours worked). All mediators are used as composite measures and are normalised to have a mean of zero and standard deviation of one, to facilitate interpretation and allow meaningful comparison of coefficients.

**Table 1:** Direct and indirect effects of WFH.

	WFH: yes = 1						WFH intensity		
Paths	Round 1	Round 1: Novice	Round 1: Established	Round 2	Round 3	Round 5	Round 2	Round 3	Round 5
	(1)	(2)	(3)	(4)	(5)	(6)	(7)	(8)	(9)
***Direct effect***									
WFH → Mental WB	0.034	0.003	0.047	-0.068	0.002	-0.012	-0.100**	-0.000	-0.100
	(1.02)	(0.07)	(1.27)	(-1.46)	(0.07)	(-0.32)	(-2.03)	(-0.00)	(-1.28)
***Indirect effects***									
WFH → WFC → Mental WB	-0.017***	-0.014***	-0.018***	-0.002	-0.006	-0.003	-0.001	-0.006	-0.002
	(-3.65)	(-3.36)	(-3.63)	(-0.33)	(-1.45)	(-0.58)	(-0.19)	(-1.44)	(-0.42)
WFH → FWC → Mental WB	0.012**	0.023***	0.008	0.010	0.015*	0.044***	0.021	0.032***	0.078***
	(2.19)	(3.47)	(1.26)	(0.66)	(1.75)	(4.07)	(1.42)	(3.29)	(4.81)
WFH → Stability → Mental WB	0.049***	0.054***	0.046***	0.020***	0.025***	0.016**	0.021***	0.031***	0.017**
	(6.95)	(7.11)	(6.31)	(2.69)	(4.07)	(2.04)	(2.65)	(4.29)	(1.99)
WFH → Resilience → Mental WB	0.017*	0.014	0.018*	0.007	0.024**	0.049***	0.015	0.018*	0.044**
	(1.85)	(1.28)	(1.82)	(0.49)	(2.54)	(3.28)	(0.90)	(1.71)	(2.54)
WFH → Networks: family/friends → Mental WB	0.010**	0.014***	0.008*						
	(2.13)	(2.70)	(1.67)						
WFH → Networks: institutional → Mental WB	0.002	0.002	0.002						
	(1.30)	(1.10)	(1.23)						
WFH → Isolation → Mental WB				-0.005	0.044***	0.063***	-0.017	0.039**	0.053***
				(-0.31)	(2.87)	(4.68)	(-0.95)	(2.42)	(3.22)
WFH → Workload → Mental WB				0.011			0.014		
				(0.83)			(1.03)		
WFH → Phys. risk → Mental WB				-0.015			-0.018		
				(-1.32)			(-1.09)		
WFH → Accommodation → Mental WB						0.013**			0.014**
						(2.14)			(2.05)

Note: Columns (1)-(6) refer to results when WFH is defined as a binary variable in terms of whether a person worked from home during the pandemic (WFH = yes), whereby columns (2) and (3) refer to round 1 and whether somebody was a WFH novice (i.e. without any prior experience of WFH) or an established WFH worker (i.e. with prior WFH experience). Columns (7)-(9) refer to results when the intensity of WFH is used instead (WFH intensity)–defined as the ratio of the total number of hours worked from home to the total number of hours worked–for rounds 2, 3 and 5, respectively. A bootstrap bias-corrected method with a 95% confidence interval was used to calculate path estimates. Robust t-statistics in parentheses

*** p<0.01

** p<0.05

* p<0.1.

Source: Living, Working and COVID-19 (Eurofound); rounds 1, 2, 3 and 5.

The results show that WFH had no significant *direct* relationship with mental well-being in any of the four survey rounds, even controlling for all indirect effects.

As concerns *indirect* effects, the results point to several important mediators; but their importance changed over the two years under consideration. As for mediators that are observable in all survey rounds, an important role is attributable to stability (of job, accommodation and income), which mediated the relationship between WFH and mental well-being in all survey rounds. Specifically, WFH was associated with greater stability, which in turn was associated with better mental well-being. This is in line with similar studies showing that workers who switched to WFH during COVID-19 reported higher perceived financial and job stability, since they could continue to earn money and ensure their jobs either with or without any further lockdowns [64]. Notably, the size of the coefficients suggests that the importance of stability decreased over time: it was the most important mediator at the beginning of the COVID-19 pandemic (round 1), whereas it played only a minor role in the last round, conducted at the beginning of 2022.

Our results point to important differences in the type of conflict between work and family domains. A positive indirect effect is observable for FWC, which stems from two negative constituent effects: WFH is associated with less FWC, which in turn is associated with better mental well-being. However, FWC was only statistically significant in round 1, when most EU member states were in their first lockdown, and in the last round, when the restrictions had already largely been relaxed. Furthermore, a comparison of coefficients suggests that FWC gained in importance over time and became a sizeable mediator when the restrictions had largely been lifted (round 5). A further distinction by previous experience of WFH suggests that the mediating role of FWC at the beginning of the COVID-19 pandemic only held for WFH novices, and was absent for established WFH workers. Conversely, WFC was a negative mediator, but was only statistically significant at the beginning of the pandemic (round 1). This stems from two opposing constituent effects. WFH was associated with greater WFC, which in turn was associated with lower mental well-being. Moreover, there are no differences according to previous WFH experience, and WFH was a negative mediator for both WFH novices and established WFH workers. The opposing effects of FWC and WFC are partly at odds with what is typically observed in the related COVID-19 literature, which finds negative effects for both FWC and WFC [65–69].

Resilience was another important positive mediator, but only one year into the pandemic (when countries were still in various stages of lockdown) and at the beginning of 2022, once the restrictions had largely been lifted (i.e. rounds 3 and 5); meanwhile it was insignificant (or only marginally significant) during the first COVID-19 year (i.e. rounds 1 and 2). As with FWC, resilience gained in importance over time and constituted a key mediator once the COVID restrictions had largely been lifted.

Other mediators that were only available for a single or a limited number of rounds also prove to have been relevant. For instance, there are important differences by type of support network. Information on support networks is only available for round 1. Specifically, networks of family and friends were relevant, in that WFH was associated with a greater importance of networks of family and friends, which in turn were associated with better mental well-being. The important role played by family and friends during the pandemic is corroborated by several studies which indicate that support from family members increased during the pandemic, especially for those with poorer mental health [70]. This mediating role of networks of family and friends was also observable for WFH novices, whereas it was only marginally significant for those who already had some prior experience of WFH. This underscores the fact that networks of family and friends were particularly important for the mental well-being of WFH novices. By contrast, institutional networks played no significant role in the mental well-being of those who worked from home.

Isolation was another highly relevant mediator, especially one year into the pandemic (when countries were again in various stages of lockdown), as well as at the beginning of 2022, when COVID restrictions had generally been lifted (i.e. rounds 3 and 5); meanwhile, it was insignificant during the first COVID-19 year (round 2). Specifically, WFH was associated with less isolation, which in turn was associated with better mental well-being. This is at odds with what is typically found in the related literature, which emphasises the negative role played by isolation and which associates that with poorer mental health among those who worked from home [e.g. 34–36] and increased depression and suicidal ideation [71]. The size of the coefficients suggests that isolation may have been the most important mediator in rounds 3 and 5; it was even more important after the COVID-19 restrictions were largely lifted (round 5).

A non-negligible role was also played by workers’ housing conditions, which was another positive mediator, but was only available for the final round. Our results suggest that WFH was related to better accommodation, which in turn was associated with better mental well-being. The importance of housing conditions for mental health in a WFH context has also been highlighted in other studies, which emphasise that better housing quality [72] and better functionality of the technology available in the home office [24] have a strong positive impact on residents’ mental health.

Finally, both workload and the physical risk of contracting COVID-19 –only available in round 2 –proved insignificant. The lack of significance for workload is consistent with [35], the longitudinal study closest to ours, while other studies have found that increased workload during the pandemic resulted in increased psychological distress [73].

There are both important similarities and certain differences when WFH intensity is used instead; this is only available for rounds 2, 3 and 5 (see columns (7)-(9)). For instance, an important difference relates to the *direct* effect of WFH: this was negatively significant, but only in the first COVID-19 year (round 2), once the restrictions of the first lockdown had been relaxed. This points to the poorer mental well-being of those who worked more intensively from home, even after controlling for all mediators.

As for the similarities, FWC, stability, resilience, isolation and housing conditions remain important positive mediators between WFH and mental well-being. However, in contrast to the findings for the binary WFH indicator, the results for WFH intensity no longer ascribe the most important role to isolation across rounds 2, 3 and 5. Instead, FWC appears to have played the most important role in terms of the size of the coefficients, especially once the COVID-19 restrictions had largely been lifted (round 5).

#### Multigroup analysis by gender: Direct and indirect effects of WFH on mental well-being

In the multigroup analysis by gender, we differentiate between male and female, but exclude the ‘other’ category, which represents only 0.23% of the final sample (or 203 persons in total) and is therefore too small to allow for a separate group to be specified and analysed. We are therefore unable to analyse and understand the challenges faced by persons who identify as ‘other’ in this context, but want to stress that mental well-being varies between the genders and responds differently to COVID-19 [74].

Using the baseline model, the *invariance of the measures across genders* was analysed first, in order to determine whether they have the same measurement properties and can therefore be compared across genders. Invariance was tested for the only latent construct used in the model, namely mental well-being. The sample means, standard deviations and correlations of the key variables by gender are displayed in S3 and S4 Tables. As is standard in the literature, we proceed stepwise and first test for configural invariance (same factor pattern/structure across groups), then for metric (weak) factorial invariance (same factor loadings across groups) and then for scalar (strong) factorial invariance (same intercepts across groups). To test each level of invariance, the difference in fit of the more constrained model is compared to that of the next, less constrained model. As mentioned above, invariance is typically evaluated using the *χ*^2^ difference test. However, since we use weights, *χ*^2^ statistics are not available, and so we not only use the SRMR and the CD, but also perform adjusted Wald tests and report p-values from the null hypothesis of (metric and scalar) factorial invariance of each construct across groups.

The results are reported in S5 Table and generally show good model fit for both males and females across all survey rounds, with all SRMRs far below the threshold of 0.08 and CDs ranging from 0.3 to 0.7. Moreover, fit statistics from the multigroup model show that the SRMR and the CD are good for all models, across all rounds: all SRMRs are below the threshold of 0.08 and all CDs are above 0.9. Both fit statistics remain robust when increasingly restrictive models are tested. Only the SRMR for round 5 is slightly above 0.08. Furthermore, results from adjusted Wald tests support metric invariance–except for round 1, for which, however, we find support for partial metric invariance [75] once the parameters for two construct items are released: namely ‘I have felt calm and relaxed’ and ‘I have felt active and vigorous’. By contrast, we fail to find support for scalar invariance, suggesting that equality of intercepts across genders cannot be supported. Since we do not conduct a comparison of latent means across genders, metric invariance suffices.

The direct and indirect effects on mental well-being of WFH obtained from the multigroup analysis, using a bootstrap bias-corrected method with a 95% confidence interval, are reported in Table 2 and Table 3. Columns (1)-(12) in Table 2 refer to results when WFH is defined as a binary variable, in terms of whether or not a person worked from home during the pandemic, with odd column numbers referring to the results for males and even column numbers to the results for females. Columns (13)-(18) in Table 3 refer to results when the frequency of WFH is used instead. Odd column numbers again refer to the findings for males and even column numbers to the findings for females.

**Table 2:** Direct and indirect effects of WFH by gender: WFH.

	WFH: yes = 1											
	Round 1		Round 1: Novice		Round 1: Established		Round 2		Round 3		Round 5	
Paths	Male	Female	Male	Female	Male	Female	Male	Female	Male	Female	Male	Female
	(1)	(2)	(3)	(4)	(5)	(6)	(7)	(8)	(9)	(10)	(11)	(12)
***Direct effect***												
WFH → Mental WB	-0.030	0.102**	-0.086	0.080*	-0.013	0.113**	-0.006	-0.119**	0.058	-0.063	-0.031	0.002
	(-0.65)	(2.47)	(-1.57)	(1.81)	(-0.26)	(2.49)	(-0.09)	(-2.02)	(1.14)	(-1.49)	(-0.63)	(0.03)
***Indirect effects***												
WFH → WFC → Mental WB	-0.012*	-0.024***	-0.011	-0.018***	-0.012*	-0.027***	-0.011	0.003	-0.010	-0.002	-0.004	0.001
	(-1.80)	(-3.84)	(-1.63)	(-3.45)	(-1.79)	(-3.86)	(-0.95)	(0.45)	(-1.41)	(-0.51)	(-0.68)	(0.10)
WFH → FWC → Mental WB	0.010	0.015**	0.020	0.025***	0.006	0.009	-0.028	0.056***	0.015	0.016*	0.056***	0.035***
	(1.03)	(2.42)	(1.62)	(3.36)	(0.65)	(1.36)	(-1.24)	(2.90)	(0.91)	(1.74)	(3.28)	(2.71)
WFH → Stability → Mental WB	0.052***	0.046***	0.057***	0.052***	0.050***	0.042***	0.017	0.025**	0.018**	0.029***	0.021**	0.012
	(4.46)	(5.58)	(4.23)	(5.67)	(4.21)	(4.94)	(1.61)	(2.34)	(2.39)	(3.59)	(1.98)	(1.10)
WFH → Resilience → Mental WB	0.030*	0.003	0.025	0.001	0.031*	0.004	-0.001	0.018	0.018	0.025**	0.057***	0.032
	(1.82)	(0.30)	(1.21)	(0.11)	(1.80)	(0.36)	(-0.04)	(1.07)	(1.06)	(2.26)	(2.81)	(1.49)
WFH → Networks: family/friends → Mental WB	0.013**	0.006	0.015*	0.012*	0.013*	0.002						
	(2.11)	(0.97)	(1.93)	(1.82)	(1.93)	(0.36)						
WFH → Networks: institutional → Mental WB	0.002	0.002	0.004	0.001	0.002	0.002						
	(0.88)	(1.23)	(1.00)	(0.88)	(0.70)	(1.25)						
WFH → Isolation → Mental WB							0.002	-0.005	0.034	0.055***	0.067***	0.057***
							(0.08)	(-0.19)	(1.63)	(2.66)	(3.41)	(2.88)
WFH → Workload → Mental WB							-0.010	0.027*				
							(-0.47)	(1.71)				
WFH → Phys. risk → Mental WB							-0.031*	0.006				
							(-1.81)	(0.33)				
WFH → Accommodation → Mental WB											0.013*	0.011
											(1.96)	(1.13)

Note: Bootstrap bias-corrected method with 95% confidence interval to calculate path estimates. Robust t-statistics in parentheses

*** p<0.01

** p<0.05

* p<0.1.

Source: Living, Working and COVID-19 (Eurofound); rounds 1, 2, 3 and 5.

**Table 3:** Direct and indirect effects of WFH by gender: WFH intensity.

	WFH intensity					
	Round 2		Round 3		Round 5	
Paths	Male	Female	Male	Female	Male	Female
	(13)	(14)	(15)	(16)	(17)	(18)
***Direct effect***						
WFH → Mental WB	-0.051	-0.153**	0.016	-0.029	-0.083	-0.065
	(-0.764)	(-2.284)	(0.275)	(-0.665)	(-1.228)	(-0.766)
***Indirect effects***						
WFH → WFC → Mental WB	-0.009	0.003	-0.011	-0.003	-0.004	0.002
	(-0.78)	(0.45)	(-1.26)	(-0.55)	(-0.63)	(0.24)
WFH → FWC → Mental WB	-0.006	0.051**	0.032	0.034***	0.106***	0.056***
	(-0.30)	(2.28)	(1.75)	(3.23)	(4.30)	(3.00)
WFH → Stability → Mental WB	0.021*	0.021*	0.022*	0.034***	0.022**	0.013
	(1.84)	(1.84)	(2.56)	(3.51)	(2.01)	(1.07)
WFH → Resilience → Mental WB	0.001	0.025	0.006	0.024*	0.066***	0.010
	(0.05)	(1.42)	(0.32)	(1.95)	(2.94)	(0.36)
WFH → Isolation → Mental WB	-0.015	-0.017	0.032	0.046**	0.063***	0.044*
	(-0.71)	(-0.63)	(1.45)	(1.98)	(3.08)	(1.86)
WFH → Workload → Mental WB	-0.005	0.030*				
	(-0.21)	(1.84)				
WFH → Phys. risk → Mental WB	-0.043*	0.012				
	(-1.81)	(0.56)				
WFH → Accommodation → Mental WB					0.016**	0.010
					(2.01)	(1.05)

Note: Bootstrap bias-corrected method with 95% confidence interval to calculate path estimates. Robust t-statistics in parentheses

*** p<0.01

** p<0.05

* p<0.1.

Source: Living, Working and COVID-19 (Eurofound); rounds 1, 2, 3 and 5.

The results reported in Table 2 show that WFH has a significant *direct* relationship with mental well-being–but only for women and only during the first COVID-19 year, when most EU economies were in their first lockdowns, or else immediately afterwards, when they were gradually reopening (rounds 1 and 2), also controlling for all indirect effects. However, the nature of the relationship differs and is initially positive, but then turns negative. This shows that during the first lockdown, women who worked from home had better mental well-being than women who did not. However, during the subsequent reopening phase, the mental well-being of women who worked from home was significantly worse than that of women who did not. Moreover, further differentiation by previous WFH experience in round 1 suggests that the initial positive direct effect was mainly observed among established female WFH workers. The positive effect during the first lockdown is likely related to the fact that women are disproportionately employed as essential and frontline workers in sectors that remained open during the lockdowns (with the associated higher risk of viral transmission and the concomitant fear of contracting COVID-19) [15–19]. WFH, by contrast, provided a safe and mentally less stressful work context for those women who could work from home, and especially for established WFH workers who did not switch to WFH totally unprepared.

As concerns *indirect* effects, the results point to important differences by gender. For example, for males who worked from home, important mediators were support from networks of family and friends (only available in round 1), physical risk of contracting COVID-19 (only available in round 2) and housing conditions (only available in round 5), although the latter two were only marginally significant. The positive network effect suggests that males who worked from home profited from their support networks of family and friends. In terms of constituent effects, our results show that males who worked from home received more support from family and friends than those who did not; among females, there was no such difference. In terms of better mental well-being, both genders benefited equally from their support networks of family and friends. Gender variations in social network features seem to lie at the heart of the differences observed: females tend to have larger and more diffuse social networks than males [76], and to have more confiding relationships–that is, they provide and receive more emotional and health-related support from multiple social ties during times of stress [77]. Male social networks are smaller and less intense than those of females, and men often report that their spouse is their only confidant [78]. Therefore, our results may indicate that males who worked from home benefited particularly from the support of their wives, whereas for females, the social support received from family and friends was equally important, whether or not they worked from home.

For females, important mediators were of both conflict concepts–WFC and FWC–but in different forms and at different stages of the pandemic (as is the case for the entire sample–see Table 1 above). The persistence of traditional gender roles and gender role attitudes with regard to home and family responsibilities helps to explain the observed gender differences in both conflict concepts [44, 79]: in most contemporary societies, men are considered to be the main breadwinners and women the caregivers [80]. Hence, in couples with children, the mother is still expected to look after the children and help with their schooling, so her job is more likely to be put on the back burner. So women have to juggle work and family, while men’s work takes priority. While FWC was a positive mediator and was relevant for the entire two years under consideration, for both WFH novices and established WFH workers WFC was a negative mediator and only mattered during the first lockdown phase (round 1). The enforced closure of schools and childcare facilities during the lockdowns and the additional childcare and homeschooling responsibilities that fell on families–and that were typically undertaken by women [45–47]–help to explain the relevance of both conflict concepts for females and the absence of any relevance for males. The positive FWC effect suggests that women who worked from home seem to have found it less stressful mentally to balance their (additional) family and work commitments than did women who still went into work while their children were stuck at home. Conversely, the negative WFC effect during the first lockdown was likely to have been the result of changing work demands in the newly mandated WFH context, which was often associated with increased workload pressure [81]; these demands were difficult to reconcile with a simultaneous increase in the amount of time spent on domestic activities, including housework, childcare and homeschooling. However, during the first lockdown, women–more so than men–cut their working hours so that they could better meet their new caregiving (including homeschooling) responsibilities [82]. Hence, the negative WFC effect among women was absent thereafter (i.e. in rounds 2, 3 and 5).

Workload was another mediator for women (only available in round 2); however, it was only marginally significant. The results suggest that women who worked from home had a greater workload than women who did not, which in turn was associated with poorer mental well-being.

Stability is the one mediator that was important for both genders throughout the two years under consideration. For both genders, stability was the most important mediator during the first lockdown (round 1)–with no differences according to previous experience of WFH–though its importance declined thereafter. The other mediators relevant for both genders were resilience and isolation, but at different stages of the pandemic. For women, resilience and isolation were relevant one year into the pandemic, when countries were again in various stages of lockdown (round 3). Meanwhile for males, both mediators mattered mainly at the beginning of 2022, when the COVID restrictions had largely been lifted (round 5).

Conversely, institutional networks were insignificant for both genders.

The findings barely change when WFH intensity is used instead (see Table 3). In some instances, however, coefficients become only marginally significant. A case in point is stability, which only mattered in 2021 for males (round 3) and in 2022 for females (round 5); during the first year of the pandemic (round 2), it was only marginally significant.

## Summary and conclusion

This paper has analysed the relationship between working from home (WFH) and mental well-being–measured by the WHO-5 Well-Being Index–and the changing role of different mediators in this relationship during the first two critical years of the COVID-19 pandemic, when governments repeatedly imposed lockdowns and enacted WFH mandates to contain the spread of the virus.

Our results point to the absence of a *direct* relationship between WFH and mental well-being in any of the four stages of the COVID-19 pandemic analysed; but we did identify several relevant–predominantly positive–mediators whose importance changed over the two years of the COVID-19 pandemic studied. This underscores the importance of the specific measures implemented during the pandemic (such as lockdowns and stages of reopening) for the relationship between WFH and mental well-being. Specifically, stability is the only mediator that was relevant over the entire two-year pandemic period analysed. It was the most important mediator during the first lockdown, but played only a minor role when many EU countries had already greatly reduced restrictions after the final lockdown in winter 2021/22. The remaining mediators were only relevant at specific stages of the pandemic: the conflicts between work and family domains–captured by WFC and FWC–were only relevant during the first lockdown; meanwhile, resilience and isolation only mattered during the second year of the pandemic and were the main mediators by the time most EU economies lifted their restrictions in the early months of 2022. Housing conditions and the support received from networks of family and friends (data on which were only available in certain survey rounds) also proved relevant; meanwhile institutional networks, workload and the physical risk of contracting COVID-19 proved irrelevant.

We find that the nature of some mediators seems to have been at odds with what is commonly found in the related literature. A case in point is isolation: WFH was associated with less isolation, which in turn was associated with better mental well-being. Since this was mainly observed in the second pandemic year–during another round of lockdowns, but especially once the COVID-19 restrictions had largely been lifted–it may indicate the effectiveness of some of the measures aimed at reducing the greater sense of loneliness and isolation felt by those who worked from home during the first lockdown. Or alternatively (and perhaps more likely) it may indicate the positive effect that stemmed from the easing of most of the restrictions. Moreover, WFH was associated with less FWC, which in turn was linked to better mental well-being; this effect can largely be explained by the differences between the genders (see below).

An important finding of our analysis is the differential role of support networks. The result suggests that the two types of network provide different resources and social capital [83], and that the closer and more intimate ties with members of the family and the circle of friends is of particular importance. It can be hard for institutional networks to establish such ties. From a policy perspective, our results suggest that in times of health crisis–like the COVID-19 pandemic–an expansion of institutional networks may fail to have any positive effect on mental well-being, particularly for those who work from home.

We also find that the relevance of the mediators differs according to previous WFH experience. Unlike established WFH workers, WFH novices benefited from both lower FWC and more helpful networks of family and friends. Hence, the lack of previous WFH experience, while it did play a role, was not in itself a cause of poorer mental well-being among WFH novices. Quite the contrary: in some important respects, WFH novices seem to have benefited. This suggests that they may have had important resources to fall back on–resources that experienced WFH workers lacked.

Our results differ by gender. We find that WFH had a significant *direct* relationship with mental well-being, but only among women and only in the first COVID-19 year. Moreover, as that first year wore on, the relationship flipped: during the first lockdown, women who worked from home had better mental well-being than women who did not; but during the subsequent reopening phase, the mental well-being of women who worked from home was significantly worse than that of women who did not. The positive effect during the first lockdown was related to the industry-specificity of lockdown measures: WFH provided a safe and mentally less stressful work context for women who could work from home (especially for established WFH workers, who did not switch to WFH totally unprepared), whereas essential and frontline workers–disproportionately comprising women–in those sectors that remained open during the lockdowns faced a greater risk of viral transmission and stronger negative mental health effects.

Mediators were also gender specific. For males who worked from home, an important mediator was support from networks of family and friends. Since a man often sees his wife as the only person he can confide in, our results may indicate that males who worked from home benefited particularly from the support of their spouses. For females who worked from home, important mediators were WFC and FWC; this was related to the adjustments they had to make in work and non-work hours in response to the enforced closure of schools and childcare facilities during the lockdowns. In this context, the positive effect of FWC that was found in the overall sample can be explained by the results for females: women who worked from home seem to have found it less mentally stressful (relatively speaking) to balance their (additional) family (including childcare and homeschooling) and work responsibilities than did women who still went into work while their children were stuck at home. The negative effect of WFC, which was limited to the first lockdown, is likely to have been the result of an increase in workload pressure in the newly mandated WFH context: women were forced to reduce their working hours, so that they could better meet their additional caregiving responsibilities; this had the effect of further extending the gender gap in working hours [82]. Hence, the negative WFC effect among females was absent from subsequent rounds.

While our findings are specific to the COVID-19 pandemic, they are also of general relevance and importance, especially as WFH appears to be here to stay: before the pandemic started, around 11% of employees in the EU27 worked from home (either usually or sometimes); this more than doubled to around 24% during the pandemic, declining slightly to around 22% in 2023 (see Eurostat [84]). As the share of those who sometimes work from home continues to grow, the future of WFH in the EU27 seems to lie in a hybrid model–a combination of working in an office environment and working from home. Importantly, our results highlight the fact that the double-edged nature of WFH needs to be considered when WFH arrangements between employers and employees are being drawn up: for instance, WFH increases stability and resilience, but at the expense of conflict between the domains of work and family. This is especially true of WFC and it particularly affects females. The implication is that conflict between the domains of work and family should be accorded high priority and that there should be a gendered approach to WFH arrangements.

## Supporting information

S1 Table Summary statistics, Cronbach’s alpha and correlations of key variables: Rounds 1 to 5. (DOCX)

S2 Table Measurement model: Rounds 1 to 5. (DOCX)

S3 Table Summary statistics and correlations of key variables: Males. (DOCX)

S4 Table Summary statistics and correlations of key variables: Females. (DOCX)

S5 Table Factorial invariance across genders. (DOCX)

## References

[1] Bailey DE, Kurland NB. A review of telework research: Findings, new directions, and lessons for the study of modern work. J. Organ. Behav. 2002;23(4):383–400. doi: 10.1002/job.144

[2] Gajendran RS, Harrison DA. The good, the bad, and the unknown about telecommuting: meta-analysis of psychological mediators and individual consequences. J. Appl. Psychol. 2007;92(6):1524–1541. doi: 10.1037/0021-9010.92.6.1524 18020794

[3] Bloom N, Liang J, Roberts J, Ying ZJ. Does working from home work? Evidence from a Chinese experiment. Q. J. Econ. 2015;130(1):165–218. doi: 10.1093/qje/qju032

[4] Dutcher EG. The effects of telecommuting on productivity: An experimental examination. The role of dull and creative tasks. J. Econ. Behav. Organ. 2012;84(1):355–363. doi: 10.1016/j.jebo.2012.04.009

[5] Kazekami S. Mechanisms to improve labor productivity by performing telework. Telecommun. Policy, 2020;44(2);101868. doi: 10.1016/j.telpol.2019.101868

[6] Song Y, Gao J. Does telework stress employees out? A study on working at home and subjective well-being for wage/salary workers. J. Happiness Stud. 2020;21(7):2649–2668. doi: 10.1007/s10902-019-00196-6

[7] Vander Elst T, Verhoogen R, Sercu M, Van den Broeck A, Baillien E, Godderis L. Not extent of telecommuting, but job characteristics as proximal predictors of work-related well-being. J. Occup. Environ. Med. 2017;59(10):E180–E186. doi: 10.1097/JOM.0000000000001132 28820860

[8] Windeler JB, Chudoba KM, Sundrup RZ. Getting away from them all: Managing exhaustion from social interaction with telework. J. Org. Behav. 2017;38(7):977–995. doi: 10.1002/job.2176

[9] Oakman J, Kinsman N, Lambert K. et al. Working from home in Australia during the COVID-19 pandemic: Cross-sectional results from the employees working from home (EWFH) study. BMJ Open 2022;12(4):e052733. doi: 10.1136/bmjopen-2021-052733 35379616 PMC8980729

[10] Kaduk A, Genadek K, Kelly EL, Moen P. Involuntary vs. voluntary flexible work: Insights for scholars and stakeholders. Community, Work & Family, 2019;22(4):412–442. doi: 10.1080/13668803.2019.1616532 PMC9455838 36090310

[11] Elbaz S, Provost Savard Y Richards JB. Teleworking and work-life balance during the COVID-19 pandemic: A scoping review. Can. Psychol. 2022;64(4):227–258. doi: 10.1037/cap0000330

[12] Hall C, Davidson L, Brooks SK, Greenberg N, Weston D. The relationship between homeworking during COVID-19 and both, mental health, and productivity: A systematic review. BMC Psy. 2023;11(188):1–19. doi: 10.1186/s40359-023-01221-3 37370153 PMC10294311

[13] Koçak O, Koçak ÖE, Younis MZ. The psychological consequences of COVID-19: Fear and the moderator effects of individuals’ underlying illness and witnessing infected friends and family. Int. J. Env. Res. Pub. He. 2021;18(4):1836. doi: 10.3390/ijerph18041836 33668655 PMC7917729

[14] Şimşir Z, Koç H, Seki T, Griffiths MD. The relationship between fear of COVID-19 and mental health problems: A meta-analysis. Death Stud. 2022;46(3):515–523. doi: 10.1080/07481187.2021.1889097 33641626

[15] Nabe-Nielsen K, Nilsson CJ, Juul-Madsen M, Bredal C, Hansen LOP, Hansen ÅM. COVID-19 risk management at the workplace, fear of infection and fear of transmission of infection among frontline employees. Occup. Environ. Med. 2021;78:248–254. doi: 10.1136/oemed-2020-106831 33077432

[16] Rosemberg MAS, Adams M, Polick C, Li WV, Dang J, Tsai JHC. COVID-19 and mental health of food retail, food service, and hospitality workers. J. Occup. Environ. Hyg. 2021;18(4–5):169–179. doi: 10.1080/15459624.2021.1901905 PMC8720174 33861938

[17] Salari N, Khazaie H, Hosseinian-Far A, Khaledi-Paveh B, Kazeminia M, Mohammadi M, et al. The prevalence of stress, anxiety and depression within front-line healthcare workers caring for COVID-19 patients: A systematic review and meta-regression. Hum. Resour. Health 2020;18(100). doi: 10.1186/s12960-020-00544-1 33334335 PMC7745176

[18] Sarfraz M, Ji X, Asghar M, Ivascu L, Ozturk I. Signifying the relationship between fear of COVID-19, psychological concerns, financial concerns and healthcare employees job performance: A mediated model. Int. J. Env. Res. Pub. He. 2022;19(5):2657. doi: 10.3390/ijerph19052657 35270350 PMC8909897

[19] Wilbiks JMP, Best LA, Law MA, Roach SP. Evaluating the mental health and well-being of Canadian healthcare workers during the COVID-19 outbreak. Healthc. Manag. For. 2021;34(4):205–210. doi: 10.1177/08404704211021109 34098760 PMC8225696

[20] Iversen MM, Norekvål TM, Oterhals K, Fadnes LT, Mæland S, Pakpour AH, et al. Psychometric properties of the Norwegian version of the fear of COVID-19 scale. Int. J. Ment. Health 2022;20(3):1446–1464. doi: 10.1007/s11469-020-00454-2 33495690 PMC7816751

[21] Kakar AS, Rauza MA, Lateef F. An empirical analysis of the mediating role of fear of COVID-19 between telecommuting and employees retention. Empl. Respons. Right. J. 2023;36:315–336. doi: 10.1007/s10672-023-09448-3 PMC10088751 40478986

[22] Choi EPH, Hui BPH, Wan EYF. Depression and anxiety in Hong Kong during COVID-19. Int. J. Env. Res. Pub. He. 2020;17(10):3740. doi: 10.3390/ijerph17103740 PMC7277420 32466251

[23] Niu Q, Nagata T, Fukutani N, Tezuka M, Shimoura K, Nagai-Tanami M, et al. Health effects of immediate telework introduction during the COVID-19 era in Japan: A cross-sectional study. PLoS ONE 2021;16(10):e0256530. doi: 10.1371/journal.pone.0256530 34624027 PMC8500427

[24] Niebuhr F, Borle P, Börner-Zobel F, Voelter-Mahlknecht S. Healthy and happy working from home? Effects of working from home on employee health and job satisfaction. Int. J. Environ. Res. Pub. He. 2022;19(3):1122. doi: 10.3390/ijerph19031122 35162145 PMC8834350

[25] Schifano S, Clark AE, Greiff S, Vögele C, D’Ambrosio C. Well-being and working from home during COVID-19. Inform. Technol. Peopl. 2021;36(5):1851–1869. doi: 10.1108/ITP-01-2021-0033

[26] Otsuka S, Ishimaru T, Nagata M, Tateishi S, Eguchi H, Tsuji M, et al. A cross-sectional study of the mismatch between telecommuting preference and frequency associated with psychological distress among Japanese workers in the COVID-19 pandemic. J. Occup. Environ. Med. 2021;63(9):e636–e640. doi: 10.1097/JOM.0000000000002318 34491971

[27] Van Zoonen W, Sivunen AE. The impact of remote work and mediated communication frequency on isolation and psychological distress. Eur. J. Work Organ. Psy. 2022;31(4):610–621. doi: 10.1080/1359432X.2021.2002299

[28] Fiorenzato E, Zabberoni S, Costa A, Cona G. Cognitive and mental health changes and their vulnerability factors related to COVID-19 lockdown in Italy. PLoS ONE 2021. doi: 10.1371/journal.pone.0246204 33503055 PMC7840042

[29] Gueguen G, Senik C. Adopting telework. The causal impact of working from home on subjective well-being in 2020. Brit. J. Ind. Rel. 2023;61(4):832–868. doi: 10.1111/bjir.12761

[30] Xiao Y, Becerik-Gerber B, Lucas G, Roll SC. Impacts of working from home during COVID-19 pandemic on physical and mental well-being of office workstation users. J. Occup. Environ. Med. 2021;63(3):181–190. doi: 10.1097/JOM.0000000000002097 33234875 PMC7934324

[31] Ekpanyaskul C, Padungtod C. Occupational health problems and lifestyle changes among novice working-from-home workers amid the COVID-19 pandemic. Saf. Health Work 2012;12(3):384–389. doi: 10.1016/j.shaw.2021.01.010 PMC7954240 33747597

[32] Felstead A, Reuschke D. Homeworking in the UK: Before and during the 2020 lockdown,” WISERD Report. Cardiff: Wales Institute of Social and Economic Research, 2020, Available for download from: https://www.regionalstudies.org/wp-content/uploads/2020/08/Homeworking-in-the-UK_Report_Final_3.pdf.

[33] Somasundram KG, Hackney A, Yung M, et al. Mental and physical health and well-being of Canadian employees who were working from home during the COVID-19 pandemic. BMC Pub. He. 2022;22:1987. doi: 10.1186/s12889-022-14349-5 36316683 PMC9619010

[34] Wels J, WielgoszewskaI B, Moltrecht B, et al. Home working and social and mental wellbeing at different stages of the COVID-19 pandemic in the UK: Evidence from 7 longitudinal population surveys. PLoS Med. 2022;20(4):e1004214. doi: 10.1371/journal.pmed.1004214 PMC10138202 37104282

[35] Wood SJ, Michaelides G, Inceoglu I, Hurren ET, Daniels K, Niven K. Homeworking, well-being and the COVID-19 pandemic: A diary study. Int. J. Environ. Res. Pub. He. 2021;18(44):7575. doi: 10.3390/ijerph18147575 34300025 PMC8307349

[36] Wielgoszewska B, Booth C, Green MJ, Hamilton OK, Wels J. Association between home working and mental health by key worker status during the Covid-19 pandemic. Evidence from four British longitudinal studies. Ind. He. 2022;60(4):345–359. doi: 10.2486/indhealth.2022-0081 35584949 PMC9453552

[37] Seddig D, Maskileyson D, Davidov E. Vaccination against COVID-19 reduces virus-related fears: Findings from a German longitudinal study. Front. Pub. He. 2022;10:878787. doi: 10.3389/fpubh.2022.878787 35968441 PMC9366712

[38] Perez-Arce F, Angrisani M, Bennett D, Darling J, Kapteyn A, Thomas K. COVID-19 vaccines and mental distress. PLoS ONE 2021;16(9):e0256406. doi: 10.1371/journal.pone.0256406 34496006 PMC8425550

[39] Koltai J, Raifman J, Bor J, McKee M, Stuckler D. COVID-19 vaccination and mental health: A difference-in-difference analysis of the understanding America study. Am. J. Prev. Med. 2022;62(5):679–687. doi: 10.1016/j.amepre.2021.11.006 35012830 PMC8674498

[40] Bodner A, Ruhl L, Barr E, Shridhar A, Skakoon-Sparling S, Card KG. The Impact of working from home on mental health: A cross-sectional study of Canadian worker’s mental health during the third wave of the COVID-19 pandemic. Int. J. Env. Res. Pub. He. 2022;19(18):11588. doi: 10.3390/ijerph191811588 36141855 PMC9517068

[41] Hornung S, Glaser J. Home-based telecommuting and quality of life: Further evidence on an employee-oriented human resource practice. Psychol. Rep. 2009;104(2):395–402. doi: 10.2466/PR0.104.2.395-402 19610467

[42] Borgman L-S, Rattay P, Lampert R. Health-related consequences of work-family conflict from a European perspective: Results of a scoping review. Front. Pub. He. 2019;7:189. doi: 10.3389/fpubh.2019.00189 PMC6629821 31338358

[43] Eddleston KA, Mulki J. Toward understanding remote workers’ management of work–family boundaries: The complexity of workplace embeddedness. Group Organ. Manag. 2017;42(3):346–387. doi: 10.1177/1059601115619548

[44] Yucel D, Chung H. Working from home, work-family conflict, and the role of gender and gender role attitudes. Community, Work & Family 2023;26(2):190–221. doi: 10.1080/13668803.2021.1993138

[45] Del Boca D, Oggero N, Profeta P, et al. Women’s and men’s work, housework and childcare, before and during COVID-19. Rev. Econ. Household 2020;18:1001–1017. doi: 10.1007/s11150-020-09502-1 PMC7474798 32922242

[46] Farré L, Fawaz Y, González L, Gaves J. How the COVID-19 lockdown affected gender inequality in paid and unpaid work in Spain. Rev. Income. Wealth. 2022;68(2):323–347. doi: 10.1111/roiw.12563

[47] Sevilla A, Smith S. Baby steps: The gender division of childcare during COVID19. Oxford Rev. Econ. Pol. 2020;36(1):S169–S186. doi: 10.1093/oxrep/graa027 PMC7499756 40504206

[48] Gualano MR, Santoro PE, Borrelli I, et al. TElewoRk-RelAted Stress (TERRA), psychological and physical strain of working from home during the COVID-19 pandemic: A systematic review. Workplace Health Saf. 2023;71(2):58–67. doi: 10.1177/21650799221119155 36382962 PMC9672980

[49] Xue B, McMunn A. Gender differences in unpaid care work and psychological distress in the UK Covid-19 lockdown. PLoS ONE 2021;16(3):e0247959. doi: 10.1371/journal.pone.0247959 33662014 PMC7932161

[50] Matthews TA, Chen L, Omidakhsh N, et al. Gender difference in working from home and psychological distress: A national survey of US employees during the COVID-19 pandemic. Ind. Health 2022;60(4):334–344. doi: 10.2486/indhealth.2022-0077 35569955 PMC9453567

[51] Griep RH, Almeida MCC, Barreto SM, et al. Working from home, work-time control and mental health: Results from the Brazilian longitudinal study of adult health (ELSA-Brasil). Front. Psychol. 2022;13:993317. doi: 10.3389/fpsyg.2022.993317 36262442 PMC9574257

[52] Nunnally JC. Psychometric Theory. McGraw Hill, New York, 1978.

[53] Bakker AB, Demerouti E, de Boer E, Schaufeli WB. Job demands and job resources as predictors of absence duration and frequency. J. Vocat. Beh. 2023a;62(2):341–356, 2003a. doi: 10.1016/S0001-8791(02)00030-1

[54] Bakker AB, Demerouti E, Taris T, Schaufeli WB, Schreurs PJG. A multi-group analysis of the job demands-resources model in four home care organizations. Int. J. Stress Manag 2003b;10(1):16–38. doi: 10.1037/1072-5245.10.1.16

[55] Demerouti E, Bakker AB, Nachreiner F, Schaufeli WB. The job demands-resources model of burnout. J. Appl. Psychol. 2001;86(3):499–512. doi: 10.1037/0021-9010.86.3.499 11419809

[56] Schaufeli WB, Bakker AB. Job demands, job resources, and their relationship with burnout and engagement: A multi-sample study. J. Org. Behav. 2004;25(3):293–315. doi: 10.1002/job.248

[57] Claes S, Vandepittem S, Clays E, Annemans L. How job demands and job resources contribute to our overall subjective well-being. Front. Psychol. 2003;14:1220263. doi: 10.3389/fpsyg.2023.1220263 PMC10394838 37539001

[58] Schaufeli WB, Taris TW. A critical review of the job demands-resources model: Implications for improving work and health. In Bauer G. F.& Hämmig O.(Eds.), Bridging occupational, organizational and public health: A transdisciplinary approach (pp. 43–68). 2014, Springer Science + Business Media. doi: 10.1007/978-94-007-5640-3_4

[59] Preacher KJ, Hayes AF. Asymptotic and resampling strategies for assessing and comparing indirect effects in multiple mediator models. Behav. Res. Methods 2008;4(3):879–891. doi: 10.3758/brm.40.3.879 18697684

[60] Hayes AF. Introduction to Mediation, Moderation, and Conditional Process Analysis: A regression-based approach. Second edition. New York: Guilford Press, 2013.

[61] Hu L-t Bentler PM. Cutoff criteria for fit indexes in covariance structural analysis: Conventional criteria versus new alternatives. Struct. Equ. Modelling 1999;6(1):1–55. doi: 10.1080/10705519909540118

[62] Wang YA, Rhemtulla M. Power analysis for parameter estimation in structural equation modeling: A discussion and tutorial. Adv. Meth. Pract. Psy. Sci. 2021;4(1). doi: 10.1177/2515245920918253

[63] Raykov T, Tisak J. Examining time-invariance in reliability in multi-wave, multi-indicator models: A covariance structure analysis approach accounting for indicator specificity. Brit. J. Math. Stat. Psy. 2004;57(2):253–263. doi: 10.1348/0007110042307267 15511307

[64] Giovanis E, Ozdamar O. Implications of COVID-19: The effect of working from home on financial and mental well-being in the UK. Int. J. He. Pol. Manag. 2022;11(9):1635–1641. doi: 10.34172/ijhpm.2021.33 33949816 PMC9808217

[65] Barriga Medina HR, Campoverde Aguirre R, Coello-Montecel D, Ochoa Pacheco P, Paredes-Aguirre MI. The influence of work–family conflict on burnout during the COVID-19 pandemic: The effect of teleworking overload. Int. J. Env. Res. Pub. He. 2021;18(19):10302. doi: 10.3390/ijerph181910302 34639602 PMC8507633

[66] Galanti T, Guidetti G, Mazzei E, Zappalà S, Toscano F. Work from home during the COVID-19 outbreak: The impact on employees’ remote work productivity, engagement, and stress. J. Occup. Environ. Med. 2021;63(7):e426–e432, 2. doi: 10.1097/JOM.0000000000002236 33883531 PMC8247534

[67] Kim HY, Hong YC, Lee N, Park J, Lee KS, Yun JY, et al. Working from home, work-life balance, and depression/anxiety among Korean workers in the COVID-19 pandemic period: A mediation analysis. J. Occup. Environm. Med. 2023;65(2):98–103. doi: 10.1097/JOM.0000000000002726 36221302 PMC9897124

[68] Lange M, Kayser I. The role of self-efficacy, work-related autonomy and work-family conflict on employee’s stress level during home-based remote work in Germany. Int. J. Env. Res. Pub. He. 2022;19(9):4955. doi: 10.3390/ijerph19094955 35564349 PMC9105450

[69] Weale V, Lambert KA, Graham M, Stuckey R, Oakman J. Do work–family conflict or family–work conflict mediate relationships between work-related hazards and stress and pain? Am. J. Ind. Med. 2023;66:780–793. doi: 10.1002/ajim.23514 37543855

[70] Al Dhaheri AS, Bataineh MF, Mohamad MN, Ajab A, Al Marzouqi A, Jarrar AH, et al. Impact of COVID-19 on mental health and quality of life: Is there any effect? A cross-sectional study of the MENA region. PLoS ONE 2021;16(3):e0249107. doi: 10.1371/journal.pone.0249107 33765015 PMC7993788

[71] Killgore WD, Cloonan SA, Taylor EC, Dailey NS. Loneliness: A signature mental health concern in the era of COVID-19. Psychiat. Res. 2020;290:113117. doi: 10.1016/j.psychres.2020.113117 32480121 PMC7255345

[72] Bower M, Buckle C, Rugel E, Donohoe-Bales A, et al. ‘Trapped’, ‘anxious’ and ‘traumatised’: COVID-19 intensified the impact of housing inequality on Australians’ mental health. Int. J. Hous. Pol. 2021;23(2):260–291. doi: 10.1080/19491247.2021.1940686

[73] Barbieri B, Balia S, Sulis I, Cois E, Cabras C, Atzara S, et al. Don’t call it smart: Working from home during the pandemic crisis. Front. Psychol. 2021;12:741585. doi: 10.3389/fpsyg.2021.741585 34659060 PMC8515044

[74] Seens H, Modarresi S, Fraser J, MacDermid JC, Walton DM, Grewal R. The role of sex and gender in the changing levels of anxiety and depression during the COVID-19 pandemic: A cross-sectional study. Women’s Health 2021;17. doi: 10.1177/17455065211062964 34844478 PMC8640979

[75] Meredith W. Measurement invariance, factor analysis and factorial invariance. Psychometrika 1993;58:525–543. doi: 10.1007/BF02294825

[76] Ang S. Life course social connectedness: age-cohort trends in social participation. Adv. Life Course Res. 2019;39:13–22, 2019. doi: 10.1016/j.alcr.2019.02.002

[77] Liebler CA, Sandefur GD. Gender differences in the exchange of social support with friends, neighbors, and co-workers at midlife. Soc. Sci. Res. 2002;31(3):364–391. doi: 10.1016/S0049-089X(02)00006-6

[78] Dykstra PA, de Jong Gierveld J. Gender and marital-history differences in emotional and social loneliness among Dutch older adults. Can. J. Aging 2004;23:141–155. doi: 10.1353/cja.2004.0018 15334814

[79] Chen G, Zhang J, Hu Y, Gao Y. Gender role attitudes and work–family conflict: A multiple mediating model including moderated mediation analysis. Front. Psychol. 2022;13. doi: 10.3389/fpsyg.2022.1032154 36619034 PMC9813485

[80] Knight CR, Brinton MC. One egalitarianism or several? Two decades of gender-role attitude change in Europe. Am J Sociol. 2017;122(5):1485–1532. doi: 10.1086/689814

[81] Wu H, Chen Y. The impact of work from home (WFH) on workload and productivity in terms of different tasks and occupations. In: Stephanidis C. et al., HCI International 2020 –Late Breaking Papers: Interaction, Knowledge and Social Media. HCII 2020. Lecture Notes in Computer Science;12427. Cham: Springer, 2020. doi: 10.1007/978-3-030-60152-2_52

[82] Collins C, Landivar LC, Ruppanner L, Scarborough WJ. COVID-19 and the gender gap in work hours. Gender. Work. Organ. 2021;28(S1):101–112. doi: 10.1111/gwao.12506 32837019 PMC7361447

[83] Putnam RD. Bowling alone: America’s declining social capital. J. Democ. 1995;6(1):65–78. doi: 10.1353/jod.1995.0002

[84] Eurostat. Employed persons working from home as a percentage of the total employment, by sex, age and professional status (%) (lfsa_ehomp).

