# Supplementary material for: Working from home and mental well-being at different stages of the COVID-19 pandemic
Source: PLoS One. 2024 Nov 13;19(11):e0312299. doi: 10.1371/journal.pone.0312299 (PMC11560032; doi:10.1371/journal.pone.0312299)
Supplement: S1 Table — (DOCX) [file pone.0312299.s001.docx]

**S1 Table. Summary statistics, Cronbach’s alpha and correlations of key variables: rounds 1 to 5**

|  | Round | Mean | Std | α/corr.  coeff^†^ | 1. | 2. | 3. | 4. | 5. | 6. | 7. | 8. | 9. | 10. | 11. | 12. | 13. | 14. | 15. |
| --- | --- | --- | --- | --- | --- | --- | --- | --- | --- | --- | --- | --- | --- | --- | --- | --- | --- | --- | --- |
| 1. WFH | R1 | 0.67 | 0.47 | - | 1 |  |  |  |  |  |  |  |  |  |  |  |  |  |  |
|  | R2 | 0.60 | 0.49 | - | 1 |  |  |  |  |  |  |  |  |  |  |  |  |  |  |
|  | R3 | 0.61 | 0.49 | - | 1 |  |  |  |  |  |  |  |  |  |  |  |  |  |  |
|  | R5 | 0.49 | 0.50 | - | 1 |  |  |  |  |  |  |  |  |  |  |  |  |  |  |
| 2. WFH: novice | R1 | 0.20 | 0.40 | - | 0.36^a^ | 1 |  |  |  |  |  |  |  |  |  |  |  |  |  |
| 3. WFH: established | R1 | 0.46 | 0.50 | - | 0.66^a^ | -0.47^a^ | 1 |  |  |  |  |  |  |  |  |  |  |  |  |
| 4. WFH intensity | R2 | 0.46 | 0.45 | - | 0.83^a^ | - | - | 1 |  |  |  |  |  |  |  |  |  |  |  |
|  | R3 | 0.45 | 0.44 | - | 0.82^a^ | - | - | 1 |  |  |  |  |  |  |  |  |  |  |  |
|  | R5 | 0.28 | 0.37 | - | 0.78^a^ | - | - | 1 |  |  |  |  |  |  |  |  |  |  |  |
| 5. Mental WB | R1 | 0.49 | 0.18 | 0.88 | 0.06^a^ | 0.00 | 0.06^a^ | - | 1 |  |  |  |  |  |  |  |  |  |  |
|  | R2 | 0.52 | 0.18 | 0.89 | 0.03^a^ | - | - | 0.02^b^ | 1 |  |  |  |  |  |  |  |  |  |  |
|  | R3 | 0.47 | 0.19 | 0.90 | 0.02^a^ | - | - | 0.01^b^ | 1 |  |  |  |  |  |  |  |  |  |  |
|  | R5 | 0.48 | 0.18 | 0.90 | 0.08^a^ | - | - | 0.05^a^ | 1 |  |  |  |  |  |  |  |  |  |  |
| 6. WFC | R1 | 0 | 1 | 0.83^†^ | 0.18^a^ | 0.04^a^ | 0.14^a^ | - | -0.24^a^ | 1 |  |  |  |  |  |  |  |  |  |
|  | R2 | 0 | 1 | 0.86^†^ | 0.17^a^ | - | - | 0.16^a^ | -0.25^a^ | 1 |  |  |  |  |  |  |  |  |  |
|  | R3 | 0 | 1 | 0.86^†^ | 0.15^a^ | - | - | 0.14^a^ | -0.26^a^ | 1 |  |  |  |  |  |  |  |  |  |
|  | R5 | 0 | 1 | 0.85^†^ | 0.11^a^ | - | - | 0.09^a^ | -0.28^a^ | 1 |  |  |  |  |  |  |  |  |  |
| 7. FWC | R1 | 0 | 1 | 0.73^†^ | -0.01^a^ | -0.02^a^ | 0.00 | - | -0.33^a^ | 0.49^a^ | 1 |  |  |  |  |  |  |  |  |
|  | R2 | 0 | 1 | 0.71^†^ | -0.01 | - | - | -0.03^a^ | -0.41^a^ | 0.47^a^ | 1 |  |  |  |  |  |  |  |  |
|  | R3 | 0 | 1 | 0.70^†^ | -0.01 | - | - | -0.03^a^ | -0.42^a^ | 0.47^a^ | 1 |  |  |  |  |  |  |  |  |
|  | R5 | 0 | 1 | 0.72^†^ | -0.07^a^ | - | - | -0.10^a^ | -0.46^a^ | 0.45^a^ | 1 |  |  |  |  |  |  |  |  |
| 8. Workload | R2 | 0 | 1 | 0.58 | 0.30^a^ | - | - | 0.26^a^ | -0.15^a^ | 0.32^a^ | 0.46^a^ | 1 |  |  |  |  |  |  |  |
| 9. Phys. risk | R2 | 0 | 1 | 0.65 | -0.37^a^ | - | - | -0.42^a^ | -0.08^a^ | 0.01 | 0.17^a^ | -0.02^b^ | 1 |  |  |  |  |  |  |
| 10. Stability | R1 | 0 | 1 | 0.65 | 0.15^a^ | 0.09^a^ | 0.07^a^ | - | 0.29^a^ | -0.12^a^ | -0.17^a^ | - | - | 1 |  |  |  |  |  |
|  | R2 | 0 | 1 | 0.56 | 0.17^a^ | - | - | 0.17^a^ | 0.31^a^ | -0.15^a^ | -0.22^a^ | 0.01 | -0.17^a^ | 1 |  |  |  |  |  |
|  | R3 | 0 | 1 | 0.62 | 0.18^a^ | - | - | 0.18^a^ | 0.32^a^ | -0.18^a^ | -0.25^a^ | - | - | 1 |  |  |  |  |  |
|  | R5 | 0 | 1 | 0.60 | 0.19^a^ | - | - | 0.16^a^ | 0.37^a^ | -0.23^a^ | -0.31^a^ | - | - | 1 |  |  |  |  |  |
| 11. Resilience | R1 | 0 | 1 | 0.73^†^ | 0.07^a^ | 0.01 | 0.06^a^ | - | 0.41^a^ | -0.17^a^ | -0.22^a^ | - | - | 0.27^a^ | 1 |  |  |  |  |
|  | R2 | 0 | 1 | 0.74^†^ | 0.05^a^ | - | - | 0.04^a^ | 0.49^a^ | -0.20^a^ | -0.25^a^ | -0.05^a^ | -0.05^a^ | 0.30^a^ | 1 |  |  |  |  |
|  | R3 | 0 | 1 | 0.76^†^ | 0.07^a^ | - | - | 0.05^a^ | 0.51^a^ | -0.22^a^ | -0.30^a^ | - | - | 0.32^a^ | 1 |  |  |  |  |
|  | R5 | 0 | 1 | 0.76^†^ | 0.09^a^ | - | - | 0.07^a^ | 0.55^a^ | -0.25^a^ | -0.35^a^ | - | - | 0.36^a^ | 1 |  |  |  |  |
| 12. NW: friends/family | R1 | 0 | 1 | 0.66 | 0.04^a^ | 0.01^b^ | 0.03^a^ | - | 0.14^a^ | 0.00 | -0.06^a^ | - | - | 0.14^a^ | 0.11^a^ | 1 |  |  |  |
| 13. NW: institutional | R1 | 0 | 1 | 0.46 | 0.06^a^ | 0.03^a^ | 0.03^a^ | - | 0.00 | 0.05^a^ | 0.03^a^ | - | - | 0.04^a^ | -0.01^b^ | -0.43^a^ | 1 |  |  |
| 14. Isolation | R2 | 0 | 1 | 0.69^†^ | -0.05^a^ | - | - | -0.02^b^ | -0.52^a^ | 0.20^a^ | 0.27^a^ | 0.09^a^ | 0.04^a^ | -0.34^a^ | -0.47^a^ | - | - | 1 |  |
|  | R3 | 0 | 1 | 0.95^†^ | -0.06^a^ | - | - | -0.03^a^ | -0.56^a^ | 0.20^a^ | 0.30^a^ | - | - | -0.34^a^ | -0.49^a^ | - | - | 1 |  |
|  | R5 | 0 | 1 | 0.71^†^ | -0.12^a^ | - | - | -0.08^a^ | -0.52^a^ | 0.23^a^ | 0.33^a^ | - | - | -0.40^a^ | -0.49^a^ | - | - | 1 |  |
| 15. Accommodation | R5 | 0 | 1 | 0.67 | -0.07^a^ | - | - | -0.06^a^ | -0.28^a^ | 0.20^a^ | 0.27^a^ | - | - | -0.32^a^ | -0.25^a^ | - | - | 0.26^a^ | 1 |

Note: † refers to the correlation coefficient in the case of two-item constructs, a p<0.001, b p<0.05.

Source: Living, Working and COVID-19 (Eurofound), own calculations.
