# Supplementary material for: Working from home and mental well-being at different stages of the COVID-19 pandemic
Source: PLoS One. 2024 Nov 13;19(11):e0312299. doi: 10.1371/journal.pone.0312299 (PMC11560032; doi:10.1371/journal.pone.0312299)
Supplement: S2 Table — (DOCX) [file pone.0312299.s002.docx]

**S2 Table. Measurement model: rounds 1 to 5**

| Item | Round 1 | | Round 2 | | Round 3 | | Round 5 | |
| --- | --- | --- | --- | --- | --- | --- | --- | --- |
|  | Unst. FL | St. FL | Unst. FL | St. FL | Unst. FL | St. FL | Unst. FL | St. FL |
| **Mental well-being** |  |  |  |  |  |  |  |  |
| I have felt cheerful and in good spirits | 1.000* | 0.778^a^ | 1.000* | 0.765^a^ | 1.000* | 0.803^a^ | 1.000* | 0.806^a^ |
| I have felt calm and relaxed | 1.004^a^ | 0.750^a^ | 1.027^a^ | 0.735^a^ | 0.995^a^ | 0.773^a^ | 1.005^a^ | 0.782^a^ |
| I have felt active and vigorous | 1.072^a^ | 0.807^a^ | 1.112^a^ | 0.814^a^ | 1.064^a^ | 0.833^a^ | 1.058^a^ | 0.835^a^ |
| I woke up feeling fresh and rested | 1.106^a^ | 0.759^a^ | 1.184^a^ | 0.776^a^ | 1.117^a^ | 0.797^a^ | 1.099^a^ | 0.786^a^ |
| I have felt cheerful and in good spirits | 1.014^a^ | 0.728^a^ | 0.982^a^ | 0.684^a^ | 1.030^a^ | 0.745^a^ | 0.937^a^ | 0.709^a^ |
| **Work-family conflict**** |  |  |  |  |  |  |  |  |
| Felt too tired after work to do some of the household jobs which needed to be done | - | 0.935^a^ | - | 0.994^a^ | - | 0.958^a^ | - | 0.970^a^ |
| Found that your job prevented you from giving the time you want to your family | - | 0.758^a^ | - | 0.738^a^ | - | 0.755^a^ | - | 0.694^a^ |
| **Family-work conflict**** |  |  |  |  |  |  |  |  |
| Found it difficult to concentrate on your job because of family | - | 0.705^a^ | - | 0.741^a^ | - | 0.722^a^ | - | 0.771^a^ |
| Found that your family responsibilities prevented you from giving the time you should to your job | - | 0.768^a^ | - | 0.735^a^ | - | 0.694^a^ | - | 0.702^a^ |
| **Stability** |  |  |  |  |  |  |  |  |
| Might lose your job in the next 3 months | 1.000* | 0.599^a^ | 1.000* | 0.557^a^ | 1.000* | 0.593 | 1.000* | 0.495^a^ |
| Have to leave accommodation within the next 6 months because you can no longer afford it | 1.300^a^ | 0.658^a^ | 1.192^a^ | 0.512^a^ | 1.381^a^ | 0.626^a^ | 1.741^a^ | 0.643^a^ |
| Your household is able to make ends meet | 0.847^a^ | 0.648^a^ | 0.792^a^ | 0.628^a^ | 0.748^a^ | 0.567^a^ | 1.068^a^ | 0.646^a^ |
| **Resilience**** |  |  |  |  |  |  |  |  |
| I find it difficult to deal with important problems that come up in my life | - | 0.739^a^ | - | 0.808^a^ | - | 0.788^a^ | - | 0.792^a^ |
| When things go wrong in my life, it generally takes me a long time to get back to normal | - | 0.771^a^ | - | 0.748^a^ | - | 0.749^a^ | - | 0.820^a^ |
| **Isolation**** |  |  |  |  |  |  |  |  |
| I feel left out of society | - | - | - | 0.682^a^ | - | 0.662^a^ | - | 0.594^a^ |
| I have felt lonely | - | - | - | 0.689^a^ | - | 0.700^a^ | - | 0.649^a^ |
| **Networks with friends/family**  Support from family/friends in each situation |  |  |  |  |  |  |  |  |
| Illness | 1.000* | 0.585^a^ | - | - | - | - | - | - |
| Advice | 1.066^a^ | 0.607^a^ | - | - | - | - | - | - |
| Job search | 0.943^a^ | 0.399^a^ | - | - | - | - | - | - |
| Feeling depressed | 0.963^a^ | 0.535^a^ | - | - | - | - | - | - |
| Childcare | 1.023^a^ | 0.443^a^ | - | - | - | - | - | - |
| Shopping | 1.077^a^ | 0.508^a^ | - | - | - | - | - | - |
| **Networks with institutions**  Support from a service provider/institution/organisation in each situation |  |  |  |  |  |  |  |  |
| Illness | 1.000* | 0.480^a^ | - | - | - | - | - | - |
| Advice | 1.066^a^ | 0.444^a^ | - | - | - | - | - | - |
| Feeling depressed | 0.681^a^ | 0.321^a^ | - | - | - | - | - | - |
| Childcare | 0.662^a^ | 0.442^a^ | - | - | - | - | - | - |
| **Workload** |  |  |  |  |  |  |  |  |
| Worked in your free time to meet work demands | - | - | 1.000* | 0.749^a^ | - | - | - | - |
| You have enough time to get the job done | - | - | 0.502^a^ | 0.495^a^ | - | - | - | - |
| Change in working hours during the COVID-19 pandemic | - | - | 0.494^a^ | 0.376^a^ | - | - | - | - |
| **Physical risk** |  |  |  |  |  |  |  |  |
| Currently at risk of contracting the COVID-19 virus because of your job? | - | - | 1.000* | 0.689^a^ | - | - | - | - |
| Currently in direct physical contact with people | - | - | 0.690^a^ | 0.471^a^ | - | - | - | - |
| Required to wear personal protective equipment to prevent the spread of COVID-19 | - | - | 0.882^a^ | 0.612^a^ | - | - | - | - |
| **Accommodation**  Thinking about your accommodation, how problematic are: |  |  |  |  |  |  |  |  |
| Lack of space in the home | - | - | - | - | - | - | 1.000* | 0.512^a^ |
| Poor insulation/energy efficiency | - | - | - | - | - | - | 0.908^a^ | 0.434^a^ |
| Poor internet connection | - | - | - | - | - | - | 0.623^a^ | 0.329^a^ |
| No access to balcony/terrace/garden | - | - | - | - | - | - | 1.069^a^ | 0.539^a^ |
| Noise from neighbours | - | - | - | - | - | - | 1.322^a^ | 0.670^a^ |
| Noise from traffic | - | - | - | - | - | - | 1.053^a^ | 0.591^a^ |

Note: * unit loading indicator constrained to 1, ** concepts only include two items, thus an identification issue emerges – hence, only the standardised factor loadings (FL) are reported, based on a fully specified measurement model, a factor loadings significant at p<0.001. Factor loadings (standardised and unstandardised) are not shown for constructs with only two items due to identification issues: work-family conflict, family-work conflict, resilience and stability.

Source: Living, Working and COVID-19 (Eurofound), own calculations.
