# Supplementary material for: Working from home and mental well-being at different stages of the COVID-19 pandemic
Source: PLoS One. 2024 Nov 13;19(11):e0312299. doi: 10.1371/journal.pone.0312299 (PMC11560032; doi:10.1371/journal.pone.0312299)
Supplement: S5 Table — (DOCX) [file pone.0312299.s005.docx]

**S5 Table. Factorial invariance across genders**

|  |  | Round 1 | Round 2 | Round 3 | Round 5 |
| --- | --- | --- | --- | --- | --- |
| Measurement model: males | SRMR | 0.057 | 0.053 | 0.060 | 0.063 |
|  | CD | 0.294 | 0.681 | 0.382 | 0.379 |
| Measurement model: females | SRMR | 0.047 | 0.050 | 0.046 | 0.062 |
|  | CD | 0.350 | 0.706 | 0.448 | 0.477 |
| Configural invariance | SRMR | 0.075 | 0.071 | 0.078 | 0.084 |
|  | CD | 0.907 | 0.958 | 0.930 | 0.929 |
| Metric invariance | SRMR | 0.075 | 0.071 | 0.078 | 0.084 |
|  | CD | 0.906 | 0.957 | 0.930 | 0.929 |
| Scalar invariance | SRMR | 0.075 | 0.071 | 0.078 | 0.084 |
|  | CD | 0.907 | 0.958 | 0.930 | 0.929 |
| Health | Metric (p-value)  Partial (p-value)  Scalar (p-value) | (0.005)  (0.196)^†^  (0.000) | (0.474)  -  (0.002) | (0.742)  -  (0.000) | (0.831)  -  (0.000) |

Note: p-values from adjusted Wald tests which test the equality of certain measurement parameters are reported in parentheses. ‘Metric’ refers to metric factorial invariance, ‘Partial’ to partial metric invariance and ‘Scalar’ to scalar factorial invariance. † released parameters are Health-2 and Health-3 (‘I have felt calm and relaxed’ and ‘I have felt active and vigorous’).

Source: Living, Working and COVID-19 (Eurofound), own calculations.
